# Enantioselective regiospecific addition of propargyltrichlorosilane to aldehydes catalyzed by biisoquinoline *N*,*N’*-dioxide

**DOI:** 10.3762/bjoc.20.255

**Published:** 2024-11-25

**Authors:** Noble Brako, Sreerag Moorkkannur Narayanan, Amber Burns, Layla Auter, Valentino Cesiliano, Rajeev Prabhakar, Norito Takenaka

**Affiliations:** 1 Department of Chemistry and Chemical Engineering, Florida Institute of Technology, 150 West University Boulevard, Melbourne, Florida 32901-6975, USA; 2 Department of Chemistry, University of Miami, 1301 Memorial Drive, Coral Gables, Florida 33146-0431, USA

**Keywords:** α-allenic alcohol, computational chemistry, Lewis base catalysis, organocatalysis, propargyltrichlorosilane

## Abstract

Distilled propargyltrichlorosilane with >99% isomeric purity was prepared for the first time, and its asymmetric catalytic regiospecific addition reaction to aldehydes was developed through a systematic catalyst structure–reactivity and selectivity relationship study. The observed catalyst structure–enantioselectivity relationship of the present allenylation reaction was found exactly opposite to that of the analogous allylation reaction. The method provided eleven α-allenic alcohols in 22–99% yield with 61:39–92:8 enantiomeric ratios. Furthermore, possible mechanisms of propargyl–allenyl isomerization of propargyltrichlorosilane were computationally investigated.

## Introduction

**Scheme 1:**
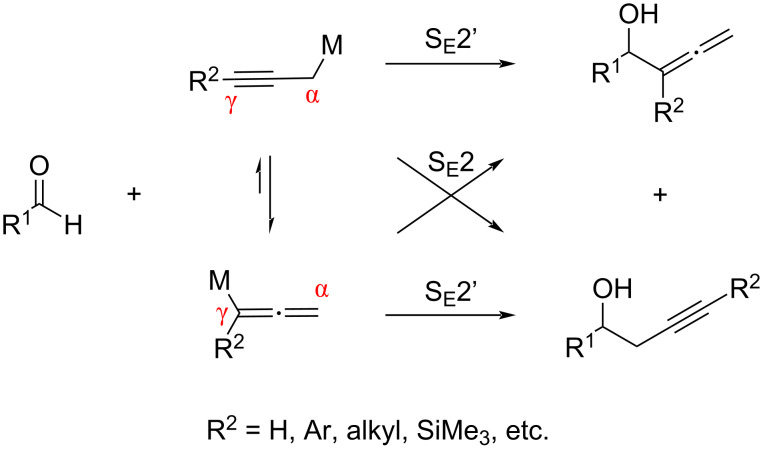
Metallotropic rearrangement and regioselectivity issues.

**Scheme 2:**
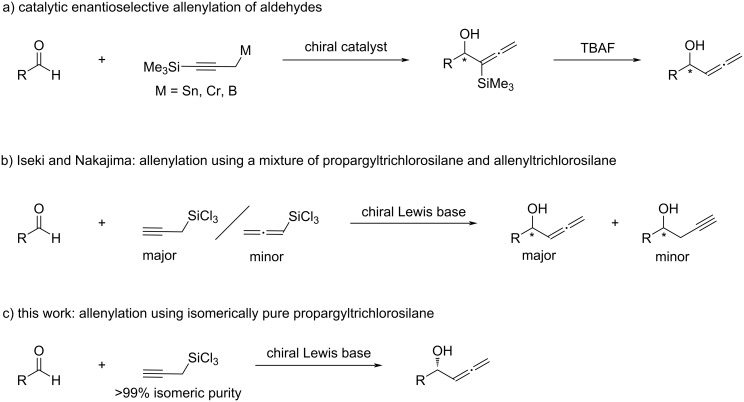
Asymmetric catalytic allenylation of aldehydes.

Enantioenriched α-allenic alcohols are an important class of chiral building blocks used for the chemical synthesis of biologically relevant molecules [1–5]. Their strength comes from the rich synthetic versatility [6–9] and biological relevance [10] of the allene functionality. Accordingly, the development of efficient access to optically active chiral α-allenic alcohols continues to be of significant interest in organic chemistry [11–13]. The asymmetric catalytic addition of allenylation reagents to aldehydes provides direct access to chiral α-allenic alcohols in an enantioenriched form [11–12]. However, such metal/metalloid reagents and the corresponding metal catalyst-bound intermediates often equilibrate between possible regioisomeric forms and can undergo both, S_E_2 and S_E_2’ addition reactions, resulting in a mixture of homopropargylic alcohols and α-allenic alcohols [14–19], the separation of which is by no means trivial [20] (Scheme 1). Nonetheless, substituents at the carbon atom indicated by γ (R^2^) of these reagents have been shown to bias the metallotropic rearrangement and/or the kinetic reactivity of the competing regioisomeric intermediates toward electrophiles [14–19]. Consequently, all reported asymmetric catalytic aldehyde allenylation methods are currently limited to metal/metalloid reagents bearing R^2^ substituents [21–34], except for the methods with propargyltrichlorosilane [35–36] (Scheme 2). Other notable asymmetric catalytic approaches to prepare α-allenic alcohols (R^2^ = H) include the Corey–Bakshi–Shibata reduction of allenyl ketones [37], enzymatic [38–40], non-enzymatic [41] kinetic resolution of racemic α-allenic alcohols, and asymmetric 1,4-difunctionalization of borylenynes by catalytic conjugative cross-coupling [42].

Allenylation reagents that require regio-controlling auxiliaries such as a trimethylsilyl group (Scheme 2a) add steps before and/or after the reaction, thus they are less efficient in terms of atom- and step-economy [21–24,33–34]. In sharp contrast, propargyltrichlorosilane is configurationally stable and only reacts through the S_E_2’ mechanism under Lewis base-catalyzed conditions [43–45], although it was reported that distillation of propargyltrichlorosilane substantially isomerizes it to the thermodynamically more stable allenyltrichlorosilane that affords undesired homopropargylic alcohols [35–36] (Scheme 2b). Furthermore, Iseki [35] and Nakajima [36] evaluated only one chiral catalyst in their independent studies (i.e., no catalyst structure–reactivity and selectivity relationship study). In this context, we became interested in investigating propargyltrichlorosilane for the development of asymmetric catalytic allenylation methods [46]. It is worthy of note that propargyltrichlorosilane is an easy-to-handle liquid and only produces innoxious NaCl and SiO_2_ as easy-to-separate inorganic byproducts upon quenching with aqueous NaOH or NaHCO_3_ solutions.

## Results and Discussion

**Scheme 3:**
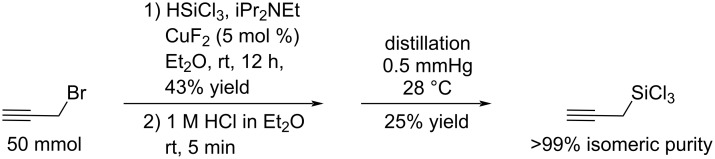
Selective preparation of propargyltrichlorosilane.

Our group recently reported that *N*,*N*-diisopropylethylamine required for the synthesis of propargyltrichlorosilane isomerized it to allenyltrichlorosilane in the absence of solvents, and that removal of the amine before the distillation significantly suppressed the isomerization [46]. In this study, however, we could not prepare isomerically pure propargyltrichlorosilane on a preparative scale from propargyl chloride and CuCl by following Kobayashi’s protocol [45] (propargyltrichlorosilane/allenyltrichlorosilane = 10:1) while we fully reproduced the reported result (2 mmol scale, propargyltrichlorosilane/allenyltrichlorosilane = >99:1). In the same report, Kobayashi also described that the combination of propargyl bromide and CuF_2_ generated propargyltrichlorosilane faster than using propargyl chloride/CuCl without attenuating the selectivity. Although both protocols reported the same result with respect to the selectivity, we decided to test whether the combination of propargyl bromide and CuF_2_ could afford isomerically pure propargyltrichlorosilane on a preparative scale. To our delight, we were able to generate propargyltrichlorosilane free of allenyltrichlorosilane on a 50 mmol scale albeit in 43% chemical yield (determined by ^1^H NMR analysis of the reaction mixture using freshly distilled anhydrous methylene chloride as an internal standard, Scheme 3). There was no sign of allenyltrichlorosilane observable by ^1^H NMR in the reaction mixture, but a trace amount of it was detected after the distillation (propargyltrichlorosilane/allenyltrichlorosilane = 200:1 by ^1^H NMR spectroscopy, see Supporting Information File 1 for details).

**Scheme 4:**
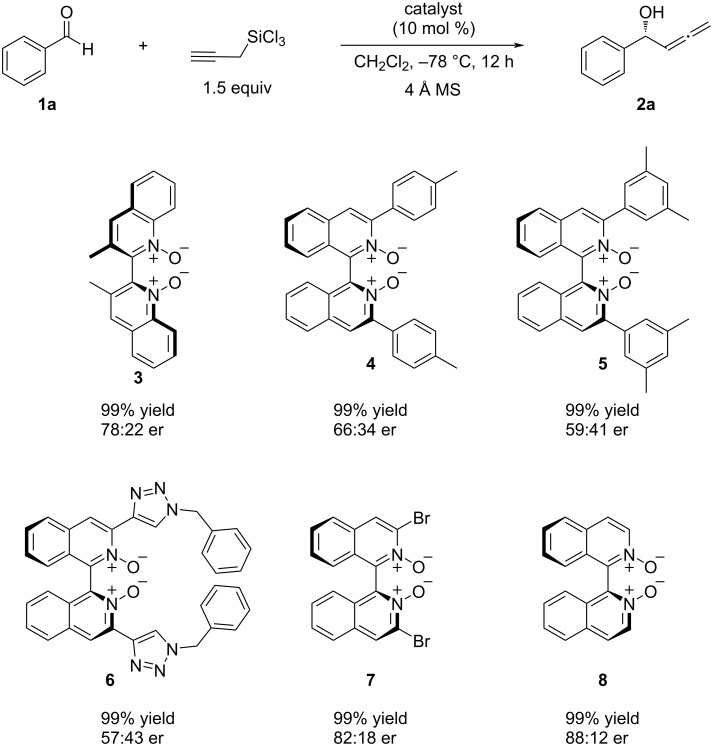
Evaluation of *C*_2_-symmetric catalysts with benzaldehyde (**1a**) as a model aldehyde. Reaction conditions: **1a** (0.1 mmol), silane (0.15 mmol), catalyst (0.01 mmol), CH_2_Cl_2_ (0.4 mL); yields were determined by ^1^H NMR spectroscopy with 1,1,2,2-tetrachloroethane as an internal standard following workup and enantiomeric ratios were determined by HPLC on a chiral stationary phase.

With distilled propargyltrichlorosilane (>99% isomeric purity) in hand, we set out on our study on the allenylation reaction of benzaldehyde (**1a**) as model aldehyde with catalyst **3** (Scheme 4). Catalyst **3** was the only catalyst previously studied by Nakajima for propargyltrichlorosilane [36]. Ever since Nakajima reported the beneficial effect of *N*,*N*-diisopropylethylamine on the reaction rate of the aldehyde allylation reaction with allyltrichlorosilane [47], it has been routinely employed in analogous chlorosilane reactions [48–55]. However, a mechanistic basis of its role on the observed rate acceleration remains elusive while it certainly functions as a scavenger of HCl inherently present in chlorosilane reagents. Given our observation regarding propargyl–allenyl isomerization as discussed above, we wanted to avoid its use. Thus, we briefly tested whether 1) it is necessary for achieving a reasonable reaction rate, and 2) if it could be substituted with 4 Å molecular sieves as an acid scavenger. The reactions with catalyst **3** in the presence of either *N*,*N*-diisopropylethylamine or 4 Å molecular sieves gave identical results (99% yield, 78:22 er), and thus we decided to proceed with molecular sieves (Scheme 4).

It is often the case that the narrower the chiral pocket of a catalyst is (i.e., less degree of conformational freedom for a bound substrate), the better is the enantioselectivity for analogous chlorosilane-mediated reactionss [47–49,51,54]. Therefore, we gradually narrowed the chiral pocket of catalysts from **3** to **4**, **5** [53], and **6** [56–57]. To our surprise, the enantioselectivity consistently decreased as the chiral pocket became narrower while the reactivity remained the same. As such, we reduced the size of the substituents that craft the chiral pocket (**7**) and found that unsubstituted catalyst **8** was the most enantioselective. This observed catalyst structure–enantioselectivity relationship is exactly opposite to that for the analogous allylation reaction reported by Nakajima [47], thus it raises a possibility that the asymmetric induction mechanism could be fundamentally different between the present allenylation with propargyltrichlorosilane and the extensively investigated allylation with allyltrichlorosilane [58–59].

**Scheme 5:**
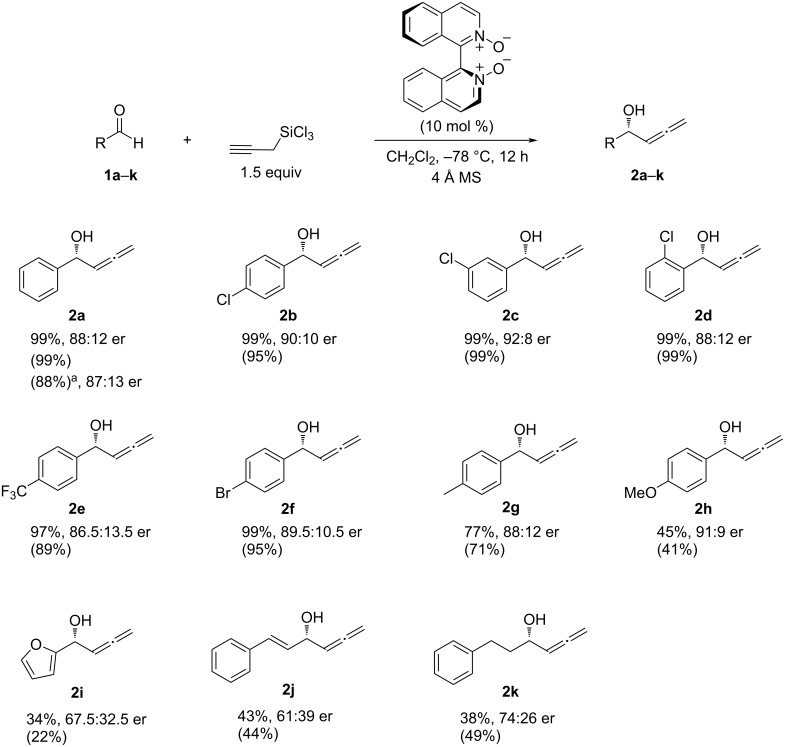
Evaluation of the extent to which (*S*)-**8** catalyzed the allenylation reaction. Reaction conditions: aldehyde **1a–k** (0.1 mmol), silane (0.15 mmol), (*S*)-**8** (0.01 mmol), CH_2_Cl_2_ (0.4 mL). Enantiomeric ratios were determined by HPLC on a chiral stationary phase, yields were determined by ^1^H NMR spectroscopy with 1,1,2,2-tetrachloroethane as an internal standard following workup and isolated yields are given in parentheses. ^a^Reaction conditions: **1a** (1.0 mmol), silane (1.5 mmol), (*S*)-**8** (0.1 mmol), CH_2_Cl_2_ (4.0 mL).

In light of the excellent reactivity and promising selectivity displayed by catalyst **8** for the model reaction, we proceeded to evaluate its ability to enantioselectively promote the allenylation of various aldehydes (Scheme 5). The catalyst tolerated all *p*-, *m*-, *o*-Cl-substituted benzaldehydes in terms of both reactivity and selectivity, affording the essentially same results as benzaldehyde (**2b**–**d**). Other electron-deficient substituents CF_3_ (**2e**) and Br (**2f**) did not adversely affect the reaction. However, electron-donating substituents (Me and MeO) on the benzene ring substantially lowered the chemical yields while they did not affect the enantioselectivity (**2g**,**h**). Likewise, electron-rich aldehydes **1i**,**j** as well as the aliphatic aldehyde **1k** provided moderate yields. These aldehydes gave substantially lower enantioselectivities than benzaldehydes, which may be attributable to that they have smaller steric demands in the vicinity of the carbonyl carbon atom than benzaldehyde. Importantly, we did not observe the corresponding homopropargylic alcohols [51] in all cases. Since this work is the first asymmetric catalysis study of isomerically pure propargyltrichlorosilane, it clearly demonstrated that this class of chiral Lewis bases regiospecifically catalyzed the addition of propargyltrichlorosilane to aldehydes, and that these catalysts did not induce the propargyl–allenyl metallotropic rearrangement albeit activating the C–Si bond. Thus, these findings underscore the importance of propargyltrichlorosilane as a regiospecific allenylation reagent and bode well for the development of new reactions.

**Figure 1:**
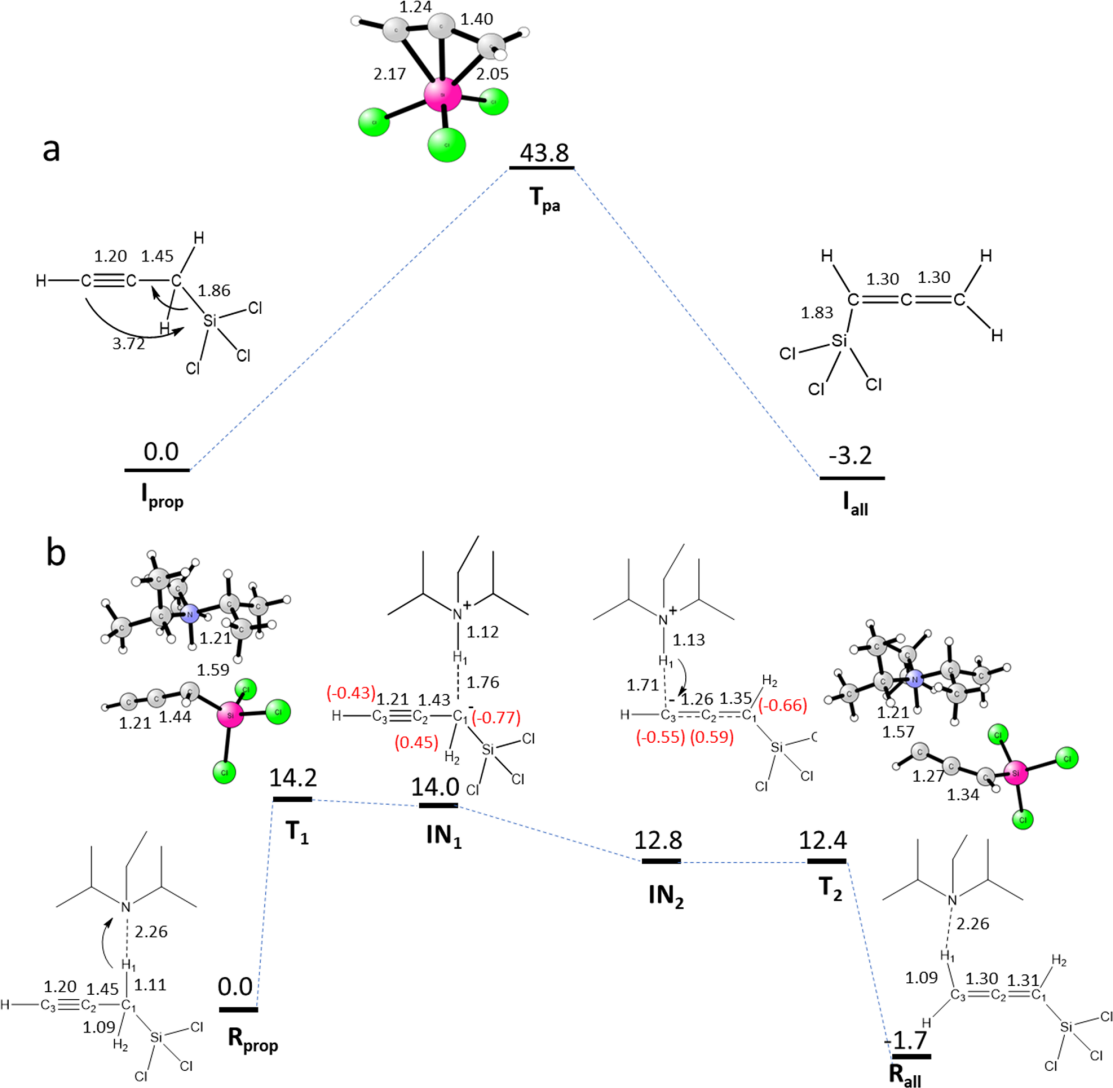
A potential energy surface (PES) for the proposed mechanism for (a) isomerization of propargyltrichlorosilane without *N*,*N*-diisopropylethylamine and (b) with *N*,*N*-diisopropylethylamine. It includes Gibbs free energies (kcal/mol) and Mulliken charges (in parentheses).

As we recently reported [46], we noticed during our initial investigation that propargyltrichlorosilane underwent isomerization to allenyltrichlorosilane in the presence of *N*,*N*-diisopropylethylamine upon standing after distillation (i.e., without solvent). Although the base-promoted propargyl–allenyl isomerization is well precedented in literature [60–61], we decided to investigate possible mechanisms of the propargyltrichlorosilane isomerization in the absence and presence of *N*,*N*-diisopropylethylamine using density functional theory (DFT) calculations (Figure 1).

According to the DFT calculations, the free propargyltrichlorosilane (**I_prop_**) can isomerize to allenyltrichlorosilane (**I_all_**) with a prohibitively high barrier of 43.8 kcal/mol (Figure 1a). This suggests that the isomerization is not energetically feasible on its own. However, in the presence of *N*,*N*-diisopropylethylamine this process can occur readily. In the reactant (**R_prop_** in Figure 1b), the nitrogen atom of *N*,*N*-diisopropylethylamine forms a hydrogen bond with the H_1_ proton of propargyltrichlorosilane and significantly activates the C_1_–H_1_ bond (1.11 Å). It is noteworthy that the nitrogen atom can also interact with the Si atom of propargyltrichlorosilane which is a stronger electrophile compared to the H_1_ atom. However, the bulky groups around nitrogen and three chlorine atoms coordinated to Si prevent a direct Si–N interaction. From **R_prop_**, the amine group of *N*,*N*-diisopropylethylamine abstracts the H_1_ proton with a barrier of 14.2 kcal/mol to form an intermediate (**IN_1_**). The intermediate **IN_1_** is unstable (endergonic by 14.0 kcal/mol) and will immediately stabilize to another intermediate (**IN_2_**) which is 1.2 kcal/mol lower in energy than **IN_1_**. The **IN_1_**→**IN_2_** transformation is driven by the redistribution of the negative charge on C_1_ in **IN_1_**. In particular, the Mulliken charge on C_1_ reduces from −0.77*e* to −0.66*e* and the charge on C_3_ increases from −0.43*e* to −0.55*e* and facilitate Coulombic interaction between C_3_ and H_1_. In the next step, from **IN_2_**, in a barrier-less process the C_3_ atom abstracts the H_1_ proton that was acquired by the N atom in the previous step to generate allenyltrichlorosilane (**R_all_**). Overall, the **R_prop_**→**R_all_** isomerization is almost thermoneutral, i.e., **R_all_** being 1.7 kcal/mol exergonic from **R_prop._** These results show that the presence of *N*,*N*-diisopropylethylamine as base makes this process more energetically feasible by substantially stabilizing the transition states and intermediates in the pathway. These results are strongly supported by the experimental data. In a previous study, Hoveyda and co-workers proposed a similar mechanism for the isomerization of alkynes to allenes catalyzed by 1,8-diazabicyclo[5.4.0]undec-7-ene [61].

## Conclusion

In this study, we prepared distilled propargyltrichlorosilane with >99% isomeric purity for the first time, developed its asymmetric catalytic regiospecific addition reaction to aldehydes via a systematic catalyst structure–reactivity and selectivity relationship study, and computationally investigated possible mechanisms of *N*,*N*-diisopropylethylamine-promoted propargyl–allenyl isomerization of propargyltrichlorosilane. The observed catalyst structure–enantioselectivity relationship of the present allenylation reaction was found exactly opposite to that of the extensively investigated analogous allylation reaction, the findings of which raises a possibility that asymmetric induction mechanisms could be fundamentally different between the two transformations. Studies directed toward a better understanding of possible transition-state structures and the design of new catalysts to improve the results are currently underway in our laboratories.

## Supporting Information

File 1 Experimental details, characterization data, spectra, and HPLC traces.

## Data Availability

All data that supports the findings of this study is available in the published article and/or the supporting information of this article.
