# Supplementary material for: Enantioselective regiospecific addition of propargyltrichlorosilane to aldehydes catalyzed by biisoquinoline N,N’-dioxide
Source: Beilstein J Org Chem. 2024 Nov 25;20:3069–76. doi: 10.3762/bjoc.20.255 (PMC11610484; doi:10.3762/bjoc.20.255)
Supplement: File 1 — Experimental details, characterization data, spectra, and HPLC traces. [file Beilstein_J_Org_Chem-20-3069-s001.pdf]

BEILSTEIN JOURNAL OF ORGANIC CHEMISTRY

Supporting Information

for

Enantioselective regiospecific addition of propargyltrichlorosilane to aldehydes catalyzed by biisoquinoline *N,N'*-dioxide

Noble Brako, Sreerag Moorkkannur Narayanan, Amber Burns, Layla Auter, Valentino Cesiliano, Rajeev Prabhakar and Norito Takenaka

Beilstein J. Org. Chem. **2024,** *20,* 3069–3076. doi:10.3762/bjoc.20.255

Experimental details, characterization data, spectra, and HPLC traces

License and Terms: This is a supporting information file under the terms of the Creative Commons Attribution License (https://creativecommons.org/licenses/by/4.0). Please note that the reuse, redistribution and reproduction in particular requires that the author(s) and source are credited and that individual graphics may be subject to special legal provisions.
The license is subject to the *Beilstein Journal of Organic Chemistry* terms and conditions: (https://www.beilstein-journals.org/bjoc/terms)

Table of contents

1. General information S1
2. Experimental procedures S2
3. Computations S10
4. References S10
5. ^{1}H NMR Spectra S12
6. HPLC traces for enantiomeric excess determination S24

1. General Information

All reactions were carried out in oven- or flame-dried glassware under an atmosphere of dry argon or nitrogen unless otherwise noted. Except as otherwise indicated, all reactions were magnetically stirred and monitored by analytical thin-layer chromatography using EMD Millipore pre-coated silica gel plates with F_{254} indicator. Visualization was accomplished by UV light (254 nm), with a combination of potassium permanganate, *p*-anisaldehyde, and/or cerium molybdate solution as an indicator. Chiral HPLC analysis was performed on Varian Polaris HPLC system with a diode array detector using analytical chiral columns (250 × 4.6 mm, L × I.D.) purchased from CHIRAL TECHNOLOGIES, INC. (CHIRALCEL® OD-H, CHIRALPAK® AD-H, and CHIRALPAK® AS-H). Flash column chromatography was performed according to the method of Still [1] using silica gel 60 (mesh 230–400) supplied by SiliCycle® Inc. Isolated yields refer to chromatographically and spectroscopically pure compounds, unless otherwise stated.

Commercial grade reagents and solvents were purchased from Sigma-Aldrich, Alfa-Aesar, Acros, Fisher, TCI, and VWR, and were used as received without further purification except as indicated below. Aldehydes were freshly distilled over CaH_2 in vacuo prior to use. Et_2O was freshly distilled over sodium/benzophenone under an atmosphere of dry nitrogen prior to use. CH_2Cl_2 was freshly distilled over CaH_2 under an atmosphere of dry nitrogen prior to use. *N*,*N*-Diisopropylethylamine was distilled over KOH under an atmosphere of dry nitrogen, stored over NaOH in a Schlenk flask, and used from there.

All ^{1}H NMR and ^{13}C NMR spectra were obtained using a Bruker 400 Ultrashield or an Oxford AS400 Spectrometer (^{1}H 400 MHz, ^{13}C 100 MHz) at ambient temperature in $CDCl_3$ purchased from Cambridge Isotope Laboratories, Inc. Chemical shifts in ^{1}H NMR spectra are reported in parts per million (ppm) respective to tetramethylsilane (δ 0.00 ppm) unless otherwise noted. The proton spectra are reported as follows δ (multiplicity, coupling constant *J*, number of protons). Multiplicities are indicated by s (singlet), d (doublet), t (triplet), q (quartet), m (multiplet), and br (broad). Chemical shifts in ^{13}C NMR spectra are reported in ppm respective to $CDCl_3$ (δ 77.0 ppm). All ^{13}C NMR spectra were recorded with complete proton decoupling. HRMS data were obtained at USF Mass Spec and Peptide Core Facility in Department of Chemistry at University of South Florida. Optical rotations were measured using a Jasco P2000 Polarimeter at 589 nm and were reported as $[\alpha]_D^{T\ °C}$, where *C* is reported in g/100 mL.

2. Experimental procedures

2.1. Synthesis and distillation of propargyltrichlorosilane

A 500 mL Schlenk round-bottomed flask equipped with a 60 mL addition funnel was charged with CuF_2 (254 mg, 2.5 mmol), freshly distilled Et_2O (80 mL), propargyl bromide (3.8 mL, 50.0 mmol), and *N*,*N*-diisopropylethylamine (17.5 mL, 100.0 mmol). The resulting mixture was cooled to 0 °C in an ice bath, and then treated dropwise with a solution of $HSiCl_3$ (11.1 mL, 110.0 mmol) in freshly distilled Et_2O (20 mL) through the addition funnel. The reaction mixture was stirred at room temperature for 12 h.

To this stirring mixture was added freshly distilled CH_2Cl_2 (3.2 mL, 50.0 mmol) as an internal standard for ^{1}H NMR analysis. Magnetic stirring was stopped to allow all solids to settle down at the bottom of the reaction flask. A small aliquot of the supernatant was transferred to a flame-dried NMR tube with a septum by a gas-tight syringe and diluted with anhydrous $CDCl_3$ (ca. 0.7 mL) that was dried over activated 4 Å molecular sieves. The ^{1}H NMR analysis of the reaction mixture indicated that 21.5 mmol of propargyltrichlorosilane (43% yield) formed and 4.8 mmol of *N*,*N*-diisopropylethylamine remained in the solution. There were no signs of allenyltrichlorosilane and propargyl bromide observable by ^{1}H NMR in the reaction mixture.

The reaction mixture was treated with a solution of HCl in Et_2O (1.0 M, 4.8 mL) and stirred for 5 min at room temperature. Magnetic stirring was stopped to allow all solids to settle down at the bottom of the reaction flask, and a small aliquot of the supernatant was analyzed by ^{1}H NMR in the same manner as above. The analysis confirmed that there was no *N*,*N*-diisopropylethylamine present in the reaction mixture, and no loss of propargyltrichlorosilane. The reaction mixture was filtered into another 500 mL Schlenk round-bottomed flask through a filter tube containing a short pad of oven-dried Celite®. The resulting clear solution was fractionally distilled as following. A short path distillation head with a 500 mL receiving flask was attached to the Schlenk flask containing the filtrate. Then, the Schlenk flask was immersed in an oil bath at 28 °C and the receiving flask was immersed in liquid nitrogen. Et_2O and other volatiles were distilled by gradually increasing the vacuum from 150 to 10 mmHg till bubbling was no longer seen at the bottom of the Schlenk flask. Then, the vacuum was released by dry nitrogen gas. The 500 mL receiving flask containing volatile distillates was replaced with a 50 mL receiving flask under an atmosphere of dry nitrogen. Propargyltrichlorosilane was then distilled into the 50 mL receiving flask immersed in liquid nitrogen in vacuo (0.5 mmHg) to give clear liquid (0.93 g, 25%). Propargyltrichlorosilane:allenyltrichlorosilane = 200:1 by ^{1}H NMR. Propargyltrichlorosilane (0.93 g, 5.4 mmol) was diluted with freshly distilled CH_2Cl_2 up to the total volume of 3.6 mL (1.5 M solution), transferred to a 10 mL Schlenk flask, and used from there. For storage, we put the 10 mL Schlenk flask in a Mason jar containing a small amount of indicating Drierite and stored the jar in the regular freezer (ca. −20 °C) to minimize the evaporation of CH_2Cl_2. So far, we have used the propargyltrichlorosilane solution in allenylation reactions up to four months without any noticeable changes. All spectral data were identical to literature values [2]. ^{1}H NMR ($CDCl_3$): δ 2.42 (d, J = 2.8 Hz, 2H), 2.11 (t, J = 2.8 Hz, 1H).

2.2. Synthesis of catalysts 3–8

(*S*)-3,3'-Dimethyl-2,2'-biquinoline *N*,*N'*-dioxide (**3**) and (*S*)-1,1'-biisoquinoline *N*,*N'*-dioxide (**8**) were prepared by following the procedures reported by Nakajima [3]. (*S*)-3,3'-Bis(4-methylphenyl)-1,1'-biisoquinoline *N*,*N'*-dioxide (**4**), (*S*)-3,3'-bis(3,5-dimethylphenyl)-1,1'-biisoquinoline *N*,*N'*-dioxide (**5**), and (*S*)-3,3'-dibromo-1,1'-

biisoquinoline *N*, *N'*-dioxide (**7**) were prepared as we previously reported [4]. (*S*)-3,3'-Bis-(1-benzyl-1*H*-1,2,3-triazole-4-yl)-1,1'-biisoquinoline *N,N'*-dioxide (**6**) was prepared as we previously reported [5].

2.3. General procedure for allenylation of aldehydes

A small test tube containing a magnetic stirring bar was charged with activated 4 Å molecular sieves powder (100 mg), (*S*)-biisoquinoline *N*,*N'*-dioxide (2.9 mg, 0.01 mmol), a solution of aldehyde in CH_2Cl_2 (1.0 M, 100 μL), and CH_2Cl_2 (200 μL). The resulting mixture was cooled to −78 °C, and then treated with a solution of propargyltrichlorosilane in CH_2Cl_2 (1.5 M, 100 μL). The reaction mixture was stirred at −78 °C for 12 h, poured into saturated aqueous $NaHCO_3$ solution (20 mL) cooled to 0 °C, and stirred at room temperature for 30 min. The resulting heterogenous mixture was filtered through a short pad of Celite® and extracted three times with CH_2Cl_2 (5 mL × 3). The combined organic layers were washed with brine, dried over Na_2SO_4, filtered, and condensed in vacuo. The resulting crude mixture was purified by flash chromatography on silica gel using EtOAc and hexanes as eluents.

(*R*)-1-Phenylbuta-2,3-dien-1-ol (2a)

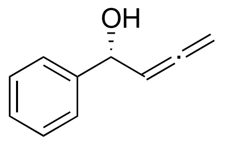

Prepared as described in general procedure using aldehyde **1a** (10.6 mg, 0.1 mmol) to give the title compound in 99% NMR yield. The crude mixture was purified by flash chromatography on silica using 15% EtOAc in hexanes as eluent to afford a pale yellow oil (14.6mg, 99%). All spectra data were identical to literature values [6,7].

^{1}H NMR ($CDCl_3$): δ 7.42-7.35 (m, 4H), 7.32-7.28 (m, 1H), 5.46 (q, J = 6.4 Hz, 1H), 5.30-5.28 (m, 1H), 4.96-4.93 (m, 2H), 2.13 (d, J = 4.0 Hz, 1H).

The (*R*)-absolute stereochemistry was assigned by HPLC analysis [9]. er = 88:12; t_R (R) 18.6 min; (S) 25.3 min, (Daicel Chiralcel® OD-H with an OD-H guard column, hexanes/2-propanol = 95/5, 0.5 mL/min, 214 nm).

$[\alpha]_D^{21}$ = +6.20 (c = 0.67, CH_2Cl_2).

(*R*)-1-(4-Chlorophenyl)buta-2,3-dien-1-ol (**2b**)

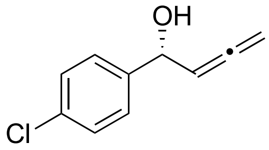

Prepared as described in general procedure using aldehyde **1b** (14.1 mg, 0.1 mmol) to give the title compound in 99% NMR yield. The crude mixture was purified by flash chromatography on silica using 15% EtOAc in hexanes as eluent to afford a viscous oil (17.1 mg, 95%). All spectra data were identical to literature values [6].

^{1}H NMR ($CDCl_3$): δ 7.36-7.33 (m, 4H), 5.41 (q, J = 6.4 Hz, 1H), 5.28-5.23 (m, 1H), 4.95-4.93 (m, 2H), 2.17(br, 1H).

The (*R*)-absolute stereochemistry was assigned by HPLC analysis [9]. er = 90:10; t_R (R) 55.7 min; (S) 59.5 min, (Daicel Chiralcel® OD-H with an OD-H guard column, hexanes/2-propanol = 99/1, 0.5 mL/min, 214 nm).

$[\alpha]_D^{20}$ = -14.60 (c = 0.76, CH_2Cl_2).

(*R*)-1-(3-Chlorophenyl)buta-2,3-dien-1-ol (**2c**)

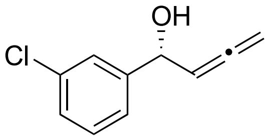

Prepared as described in general procedure using aldehyde **1c** (14.1 mg, 0.1 mmol) to give the title compound in 99% NMR yield. The crude mixture was purified by flash chromatography on silica using 15% EtOAc in hexanes as eluent to afford a viscous oil (17.9 mg, 99%). All spectra data were identical to literature values [7].

^{1}H NMR ($CDCl_3$): δ 7.41 (s, 1H), 7.30-7.26 (m, 3H), 5.42 (q, J = 6.4 Hz, 1H), 5.27-5.25 (m, 1H), 4.98-4.96 (m, 2H), 2.13 (d, J = 4.0 Hz, 1H).

The (*R*)-absolute stereochemistry was assigned by HPLC analysis [9]. er = 92:8;

t_R (R) 18.0 min; (S) 19.2 min, (Daicel Chiralcel® OD-H with an OD-H guard column, hexanes/2-propanol = 95/5, 0.5 mL/min, 214 nm).

$[\alpha]_D^{21}$ = -17.51 (c = 0.80, CH_2Cl_2).

(*R*)-1-(2-Chlorophenyl)buta-2,3-dien-1-ol (**2d**)

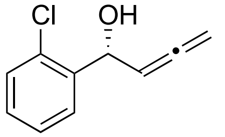

Prepared as described in general procedure using aldehyde **1d** (14.1 mg, 0.1 mmol) to give the title compound in 99% NMR yield. The crude mixture was purified by flash chromatography on silica using 15% EtOAc in hexanes as eluent to afford a viscous oil (18 mg, 99%). All spectra data were identical to literature values [8].

^{1}H NMR ($CDCl_3$): δ 7.58 (dd, J = 7.6, 1.2 Hz, 1H), 7.36-7.28 (m, 2H) 7.25-7.22 (m, 1H), 5.70-5.64 (m, 1H), 5.48 (q, J = 6.4 Hz, 1H), 4.94 (dd, J = 6.4, 2.8 Hz, 2H), 2.32 (d, J = 4.4 Hz, 1H).

The (*R*)-absolute stereochemistry was assigned by HPLC analysis [8]. er = 88:12;

t_R (R) 47.2 min; (S) 54.9 min, (Daicel Chiralcel® OD-H with an OD-H guard column, hexanes/2-propanol = 99/1, 0.5 mL/min, 214 nm).

$[\alpha]_D^{21}$ = +14.56 (c = 0.85, CH_2Cl_2).

(*R*)-1-(4-(Trifluoromethyl)phenyl)buta-2,3-dien-1-ol (**2e**)

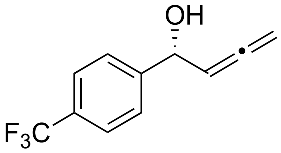

Prepared as described in general procedure using aldehyde **1e** (17.4 mg, 0.1 mmol) to give the title compound in 97% NMR yield. The crude mixture was purified by flash chromatography on silica using 15% EtOAc in hexanes as eluent to afford a viscous oil (19 mg, 89%). All spectra data were identical to literature values [8].

^{1}H NMR ($CDCl_3$): δ 7.62 (d, J = 8.0 Hz, 2H), 7.52 (d, J = 8.4 Hz, 2H), 5.41 (q, J = 6.4 Hz, 1H), 5.37-5.32 (m, 1H), 4.97-4.95 (m, 2H), 2.24 (d, J = 3.6 Hz, 1H).

The (*R*)-absolute stereochemistry was assigned by HPLC analysis [8]. er = 86.5:13.5;

t_R (R) 32.5 min; (S) 36.5 min, (Daicel Chiralpak® AD-H with an AD-H guard column, hexanes/2-propanol = 98/2, 0.5 mL/min, 214 nm).

$[\alpha]_D^{21}$ = -7.53 (c = 0.6, CH_2Cl_2).

(*R*)-1-(4-Bromophenyl)buta-2,3-dien-1-ol (2f)

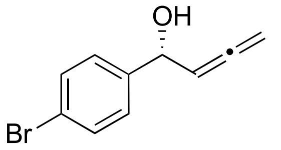

Prepared as described in general procedure using aldehyde **1f** (18.5 mg, 0.1 mmol) to give the title compound in 99% NMR yield. The crude mixture was purified by flash chromatography on silica using 15% EtOAc in hexanes as eluent to afford a viscous oil (21.3 mg, 95%). All spectra data were identical to literature values [6].

^{1}H NMR ($CDCl_3$): δ 7.49 (d, J = 8.4 Hz, 2H), 7.28 (d, J = 8.4 Hz, 2H), 5.39 (q, J = 6.4 Hz, 1H), 5.27-5.22 (m, 1H), 4.96-4.93 (m, 2H), 2.15 (d, J = 3.6 Hz, 1H).

The (*R*)-absolute stereochemistry was assigned by HPLC analysis [8]. er = 89.5:10.5; t_R (R) 79.5 min; (S) 88.9 min, (Daicel Chiralpak® AD-H with an AD-H guard column, hexanes/2-propanol = 99/1, 0.5 mL/min, 214 nm).

$[\alpha]_D^{21}$ = +6.16 (c = 0.64, CH_2Cl_2).

***(R)*-1-(*p*-Tolyl)buta-2,3-dien-1-ol** (**2g**)

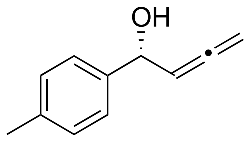

Prepared as described in general procedure using aldehyde **1g** (12.0 mg, 0.1 mmol) to give the title compound in 77% NMR yield. The crude mixture was purified by flash chromatography on silica using 20% EtOAc in hexanes as eluent to afford a viscous oil (11.4 mg, 71%). All spectra data were identical to literature values [6].

^{1}H NMR ($CDCl_3$): δ 7.30 (d, J = 8.0 Hz, 2H), 7.17 (d, J = 8.0 Hz, 2H), 5.43 (q, J = 6.4 Hz, 1H), 5.27-5.22 (m, 1H), 4.95-4.92 (m, 2H), 2.35 (s, 3H), 2.10 (d, J = 4 Hz, 1H).

The (*R*)-absolute stereochemistry was assigned by HPLC analysis [9]. er = 88:12; t_R (R) 21.4 min; (S) 24.2 min, (Daicel Chiralpak® AD-H with an AD-H guard column, hexanes/2-propanol = 95/5, 0.5 mL/min, 214 nm).

$[\alpha]_D^{20}$ = -1.25 (c = 0.50, CH_2Cl_2).

(*R*)-1-(4-Methoxyphenyl)buta-2,3-dien-1-ol (2h)

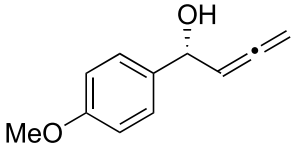

Prepared as described in general procedure using aldehyde **1h** (13.6 mg, 0.1 mmol) to give the title compound in 45% NMR yield. The crude mixture was purified by flash chromatography on silica using 20% EtOAc in hexanes as eluent to afford a pale yellow oil (7.3 mg, 41%). All spectra data were identical to literature values [6].

^{1}H NMR ($CDCl_3$): δ 7.33 (d, *J* = 8.8, 2H), 6.90 (d, *J* = 8.8, 2H), 5.44 (q, *J* = 6.4, 1H), 5.27-5.21 (m, 1H), 4.95-4.92 (m, 2H), 3.81 (s, 3H), 2.06 (d, *J* = 4 Hz, 1H).

The (*R*)-absolute stereochemistry was assigned by HPLC analysis [9]. er = 91:9;

t_R (R) 26.0 min; (S) 33.9 min, (Daicel Chiralpak® OD-H with an OD-H guard column, hexanes/2-propanol = 95/5, 0.5 mL/min, 214 nm).

$[\alpha]_D^{20}$ = +20.44 (c = 0.32, CH_2Cl_2).

***(R)*-1-(Furan-2-yl)buta-2,3-dien-1-ol (2i)**

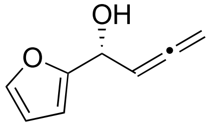

Prepared as described in general procedure using aldehyde **1i** (9.6 mg, 0.1 mmol) to give the title compound in 34% NMR yield. The crude mixture was purified by flash chromatography on silica using 20% EtOAc in hexanes as eluent to afford a viscous oil (3 mg, 22%). All spectra data were identical to literature values [9].

^{1}H NMR ($CDCl_3$) δ 7.41 (br s, 1H), 6.35-6.34 (m, 1H), 6.31 (d, *J* = 3.2 Hz, 2H), 5.54 (q, *J* = 6.4 Hz, 1H), 5.30-5.25 (m, 1H), 4.99-4.97 (m, 2H), 2.16 (d, *J* = 5.6 Hz, 1H).

The (*R*)-absolute stereochemistry was assigned by HPLC analysis [9]. er = 67.5:32.5;

t_R (S) 32.5 min; (R) 27.0 min, (Daicel Chiralcel® OD-H with an OD-H guard column, hexanes/2-propanol = 95/5, 0.5 mL/min, 214 nm).

$[\alpha]_D^{21}$ = +17.3 (c = 0.10, CH_2Cl_2).

(*S,E*)-1-Phenylhexa-1,4,5-trien-3-ol (**2j**)

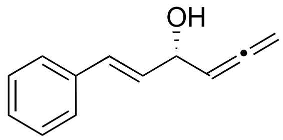

Prepared as described in general procedure using aldehyde **1j** (13.2 mg, 0.1 mmol) to give the title compound in 43% NMR yield. The crude mixture was purified by flash chromatography on silica using 20% EtOAc in hexanes as eluent to afford a viscous oil (7.5 mg, 44%). All spectra data were identical to literature values [6].

^{1}H NMR ($CDCl_3$) δ 7.40 (d, *J* = 7.2 Hz, 2H), 7.32 (t, *J* = 7.2 Hz, 2H), 7.26 (t, *J* = 5.2 Hz, 1H), 6.65 (d, *J* = 16.0 Hz, 1H), 6.28 (dd, *J* = 16.0, 6.4 Hz, 1H), 5.38 (q, *J* = 6.4 Hz, 1H), 4.94 (dd, *J* = 6.4, 2.4 Hz, 2H), 4.90-4.84 (m,1H), 1.89 (d, J = 4.4 Hz, 1H).

The (*S*)-absolute stereochemistry was assigned by HPLC analysis [9]. er = 61:39;

t_R (S) 13.0 min; (R) 14.5 min, (Daicel Chiralpak® AS-H with an AS-H guard column, hexanes/2-propanol = 90/10, 0.5 mL/min, 214 nm).

$[\alpha]_D^{21}$ = +13.29 (c = 0.23, CH_2Cl_2).

(*S*)-1-Phenylhexa-4,5-dien-3-ol (**2k**)

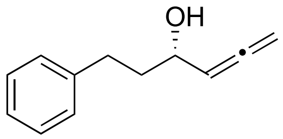

Prepared as described in general procedure using aldehyde **1k** (13.4 mg, 0.1 mmol) to give the title compound in 38% NMR yield. The crude mixture was purified by flash chromatography on silica using 15% EtOAc in hexanes as eluent to afford a viscous oil (8.6 mg, 49%). All spectra data were identical to literature values [6].

^{1}H NMR ($CDCl_3$) δ 7.27-7.17 (m, 5H), 5.28 (q, *J* = 6.4 Hz, 1H), 4.89 (dd, *J* = 6.4, 2.4 Hz, 2H), 4.25-4.16 (m,1H), 2.78-2.7 (m, 2H), 1.93-1.87 (m, 2H), 1.67 (d, *J* = 4.4 Hz, 1H).

The (*S*)-absolute stereochemistry was assigned by HPLC analysis [9]. er = 74:26;

t_R (S) 23.4 min; (R) 34.8 min, (Daicel Chiralcel® OD-H with an OD-H guard column, hexanes/2-propanol = 95/5, 0.5 mL/min, 214 nm).

$[\alpha]_D^{21}$ = +5.97 (c = 0.33, CH_2Cl_2).

3. Computations

All calculations were performed using the Gaussian 16 software package [10]. All structures were optimized using the M06-2X [11] functional and 6-311G(d) [12] basis set as implemented in Gaussian 16. Hessians were calculated with the same level of theory as the optimizations. The final energies were further improved by performing single point calculations with 6-311++g(d,p) [12,13] basis set on optimized structures. To simulate experimentally used *N*,*N*-diisopropylethylamine solvent, diethylamine was used in self-consistent reaction field-SMD implicit solvent model (SCRF-SMD) [14]. The dispersion corrections were included by performing single point calculations using Grimme D3 function [15]. Zero-point vibrational (unscaled), thermal (at 298.15 K and 1 atm), solvent, dispersion, and entropy corrections (at 298.15 K) were added to the final energies of the optimized structures.

4. References

1. Still, W. C., Kahn, M., Mitra, A., *J. Org. Chem.* **1978**, 43, 2923-2925.
2. Iseki, K., Kuroki, Y., Kobayashi, Y. *Tetrahedron: Asymmetry*, **1998**, *9*, 2889-2894.
3. Nakajima, M., Saito, M., Shiro, M., Hashimoto, S. *J. Am. Chem. Soc.* **1998**, *120*, 6419-6420.
4. Reep, C., Morgante, P., Peverati, R., Takenaka, N. *Org. Lett.* **2018**, *20*, 5757-5761.
5. Sun, S., Reep, C., Zhang, C., Captain, B., Peverati, R., Takenaka, N. *Tetrahedron Letters*, **2021**, *81*, 153338.
6. Reddy, L. R., *Chem Commun*., **2012**, *48*, 9189-9191.
7. Schneider, U., Sugiura, M., Kobayashi, S. *Tetrahedron*, **2006**, *62*, 496-502.
8. Zhang, T., Zhu, C. *Synlett.*, **2024**, *35*, 1170-1174.
9. Li, W., Lin, Z., Chen, L., Tian, X., Wang, Y., Huang, S.-H., Hong, R., *Tetrahedron Letters*, **2016**, *57*, 603-606.
10. Frisch, M.J., et al., *Gaussian 16 Rev. C.01*. 2016: Wallingford, CT.
11. Zhao, Y. and D.G. Truhlar, *The M06 suite of density functionals for main group thermochemistry, thermochemical kinetics, noncovalent interactions, excited states, and transition elements: two new functionals and systematic testing of four*

M06-class functionals and 12 other functionals. Theoretical chemistry accounts, 2008. **120**: p. 215-241.

12. Krishnan, R., et al., *Self-consistent molecular orbital methods. XX. A basis set for correlated wave functions.* The Journal of chemical physics, 1980. **72**(1): p. 650-654.
13. Clark, T., et al., *Efficient diffuse function-augmented basis sets for anion calculations. III. The 3-21+ G basis set for first-row elements, Li–F.* Journal of Computational Chemistry, 1983. **4**(3): p. 294-301.
14. Cances, E., B. Mennucci, and J. Tomasi, *A new integral equation formalism for the polarizable continuum model: Theoretical background and applications to isotropic and anisotropic dielectrics.* The Journal of chemical physics, 1997. **107**(8): p. 3032-3041.
15. Grimme, S., S. Ehrlich, and L. Goerigk, *Effect of the damping function in dispersion corrected density functional theory.* Journal of computational chemistry, 2011. **32**(7): p. 1456-1465.

03-27-2024_propargyltrichlorosilane

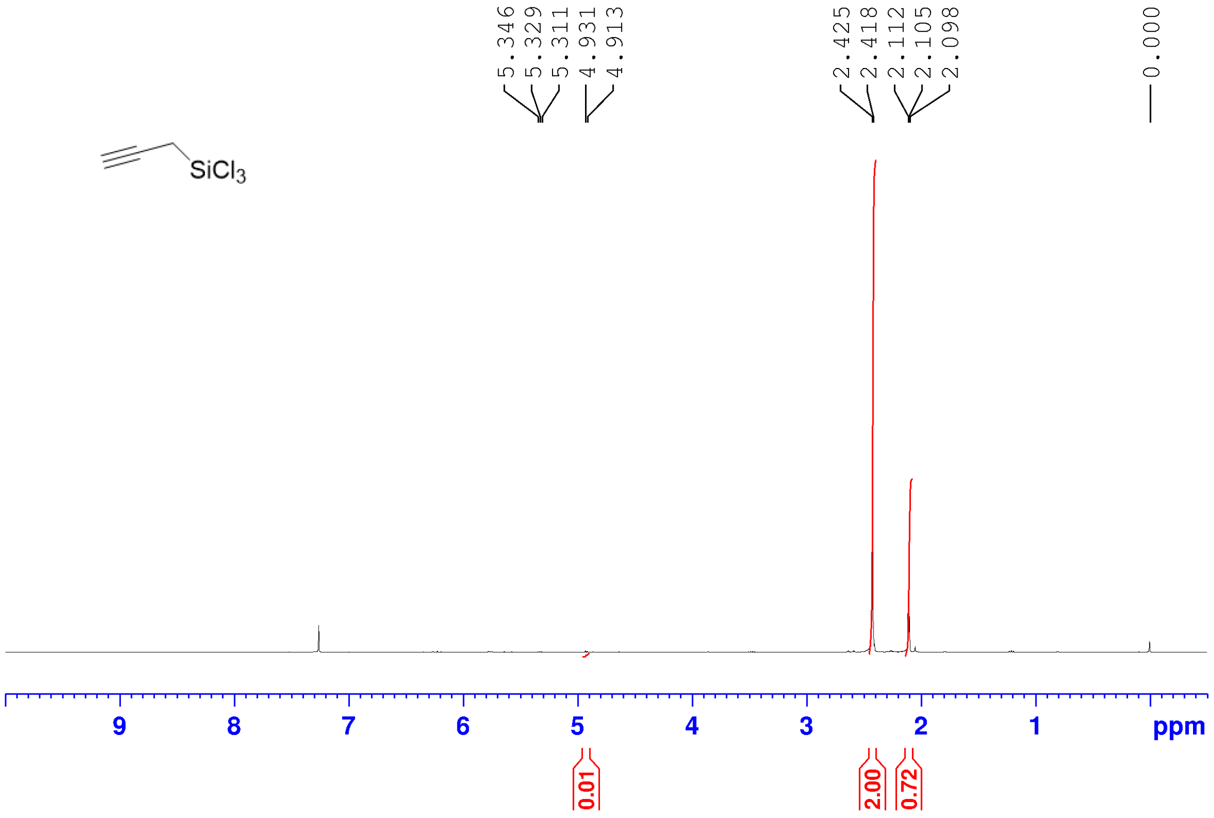


```
Current Data Parameters
NAME          Mar27-2024
EXPNO                 10
PROCNO                 1

F2 - Acquisition Parameters
Date_           20240327
Time               18.50
INSTRUM            spect
PROBHD     5 mm PABBO BB-
PULPROG             zg30
TD                 65536
SOLVENT            CDCl3
NS                     8
DS                     2
SWH             8278.146 Hz
FIDRES          0.126314 Hz
AQ             3.9583745 sec
RG                   256
DW                60.400 usec
DE                  6.50 usec
TE                 297.1 K
D1            1.00000000 sec
TD0                    1

======== CHANNEL f1 ========
NUC1                  1H
P1                 11.60 usec
PL1                    0 dB
SFO1         400.1324710 MHz

F2 - Processing parameters
SI                 32768
SF           400.1300103 MHz
WDW                   EM
SSB                    0
LB                  0.30 Hz
GB                     0
PC                  1.00
```

NB-I 172 isolated

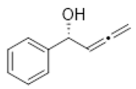

2a

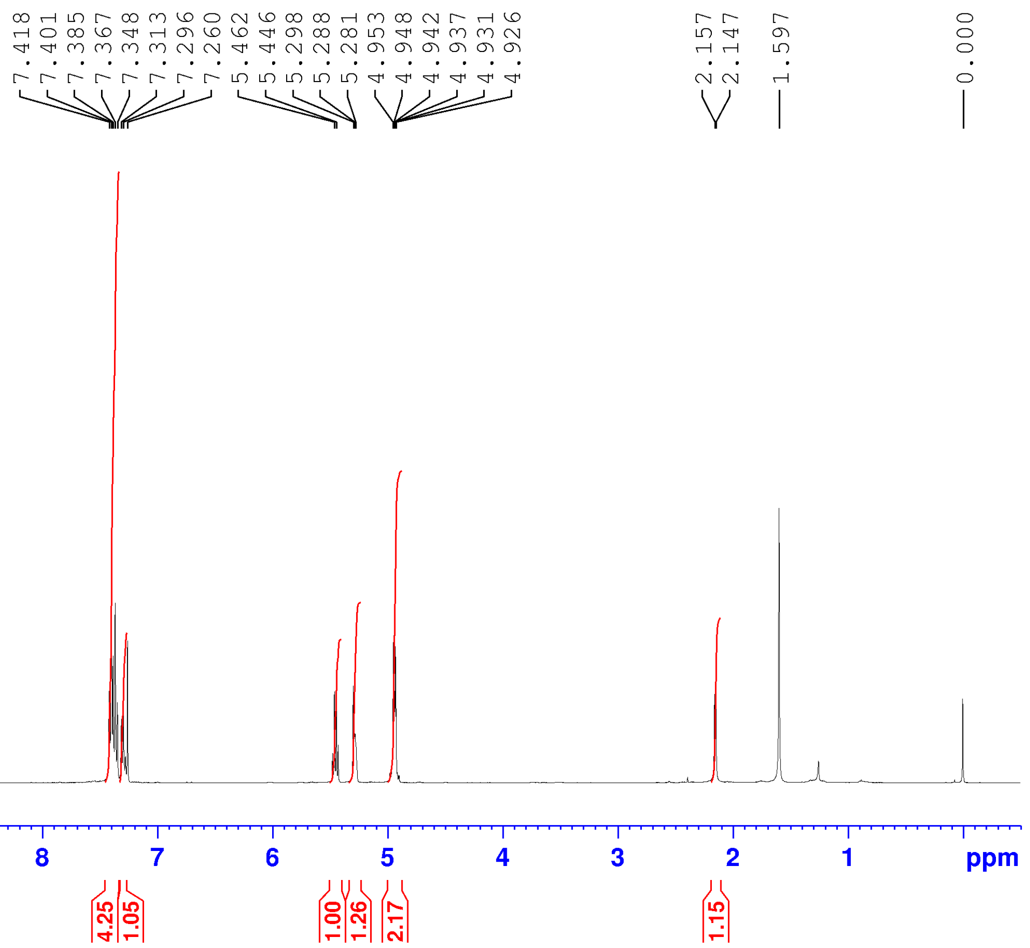


```
Current Data Parameters
NAME            May07-2024
EXPNO                    2
PROCNO                   1

F2 - Acquisition Parameters
Date_             20240507
Time                 16.32
INSTRUM              spect
PROBHD     5 mm TBI 1H/31
PULPROG               zg30
TD                   65536
SOLVENT              CDCl3
NS                      16
DS                       2
SWH               8278.146 Hz
FIDRES            0.126314 Hz
AQ               3.9583745 sec
RG                     512
DW                  60.400 usec
DE                    6.50 usec
TE                   300.0 K
D1              1.00000000 sec
TD0                      1

======== CHANNEL f1 ========
NUC1                    1H
P1                   18.75 usec
PL1                   0.50 dB
SFO1           400.1324710 MHz

F2 - Processing parameters
SI                   32768
SF             400.1300098 MHz
WDW                     EM
SSB                      0
LB                    0.30 Hz
GB                       0
PC                    1.00
```

NB-I 174 isolated

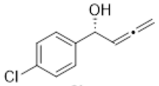

2b

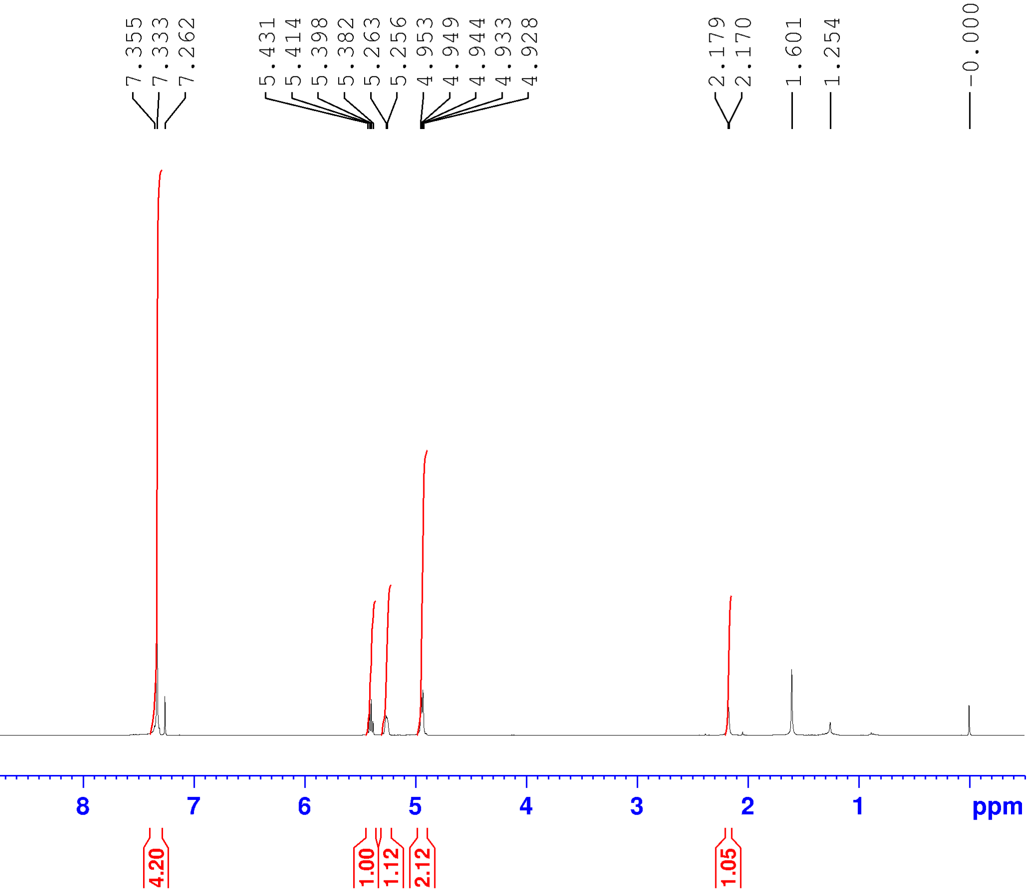


```
Current Data Parameters
NAME            May08-2024
EXPNO                    3
PROCNO                   1

F2 - Acquisition Parameters
Date_             20240508
Time                 16.12
INSTRUM              spect
PROBHD    5 mm TBI 1H/31
PULPROG               zg30
TD                   65536
SOLVENT              CDCl3
NS                      16
DS                       2
SWH               8278.146 Hz
FIDRES            0.126314 Hz
AQ               3.9583745 sec
RG                     512
DW                  60.400 usec
DE                    6.50 usec
TE                   300.0 K
D1              1.00000000 sec
TD0                      1

======== CHANNEL f1 ========
NUC1                    1H
P1                   18.75 usec
PL1                   0.50 dB
SFO1           400.1324710 MHz

F2 - Processing parameters
SI                   32768
SF             400.1300093 MHz
WDW                     EM
SSB                      0
LB                    0.30 Hz
GB                       0
PC                    1.00
```

NB-I 182 isolated

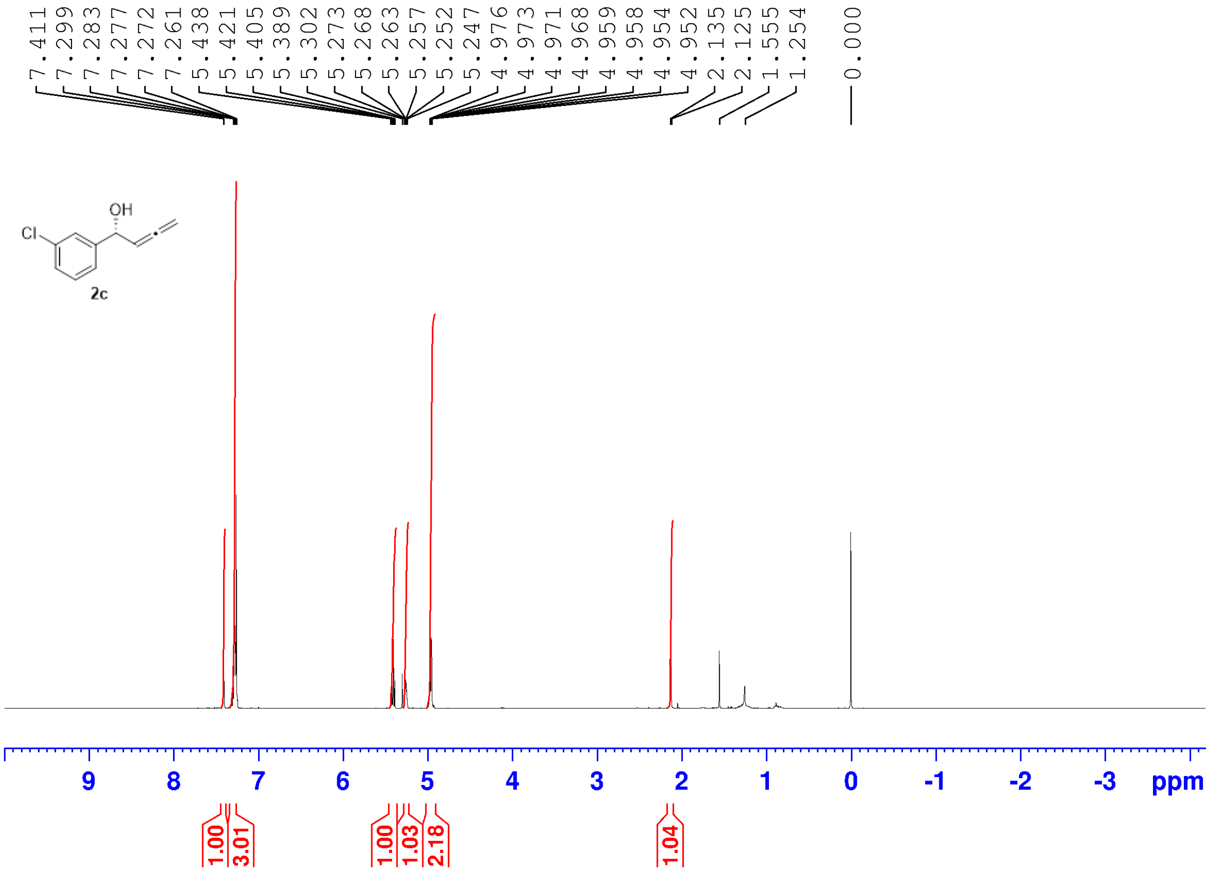


```
Current Data Parameters
NAME          May11-2024
EXPNO                  4
PROCNO                 1

F2 - Acquisition Parameters
Date_           20240511
Time               14.21
INSTRUM            spect
PROBHD   5 mm TBI 1H/31
PULPROG             zg30
TD                 65536
SOLVENT            CDCl3
NS                    16
DS                     2
SWH            8278.146 Hz
FIDRES         0.126314 Hz
AQ            3.9583745 sec
RG                 812.7
DW                60.400 usec
DE                  6.50 usec
TE                 300.0 K
D1            1.00000000 sec
TD0                    1

======== CHANNEL f1 ========
NUC1                  1H
P1                 18.75 usec
PL1                 0.50 dB
SFO1         400.1324710 MHz

F2 - Processing parameters
SI                 32768
SF           400.1300092 MHz
WDW                   EM
SSB                    0
LB                  0.30 Hz
GB                     0
PC                  1.00
```

NB-I 180 isolated

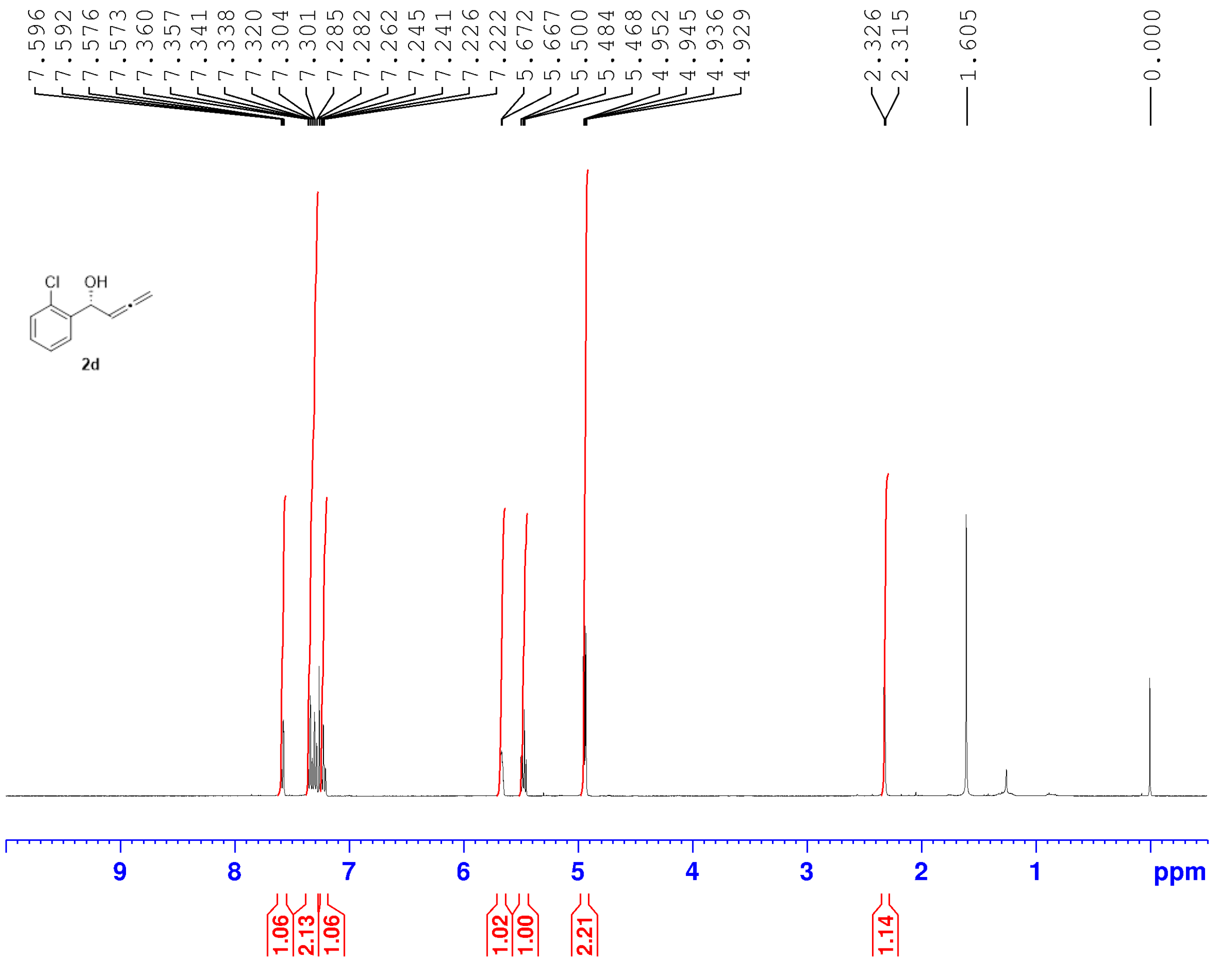


```
Current Data Parameters
NAME             May10-2024
EXPNO                     7
PROCNO                    1

F2 - Acquisition Parameters
Date_              20240510
Time                  21.38
INSTRUM               spect
PROBHD    5 mm TBI 1H/31
PULPROG                zg30
TD                    65536
SOLVENT               CDCl3
NS                       16
DS                        2
SWH                8278.146 Hz
FIDRES             0.126314 Hz
AQ                3.9583745 sec
RG                      512
DW                   60.400 usec
DE                     6.50 usec
TE                    300.0 K
D1               1.00000000 sec
TD0                       1

======== CHANNEL f1 ========
NUC1                     1H
P1                    18.75 usec
PL1                    0.50 dB
SFO1            400.1324710 MHz

F2 - Processing parameters
SI                    32768
SF              400.1300089 MHz
WDW                      EM
SSB                       0
LB                     0.30 Hz
GB                        0
PC                     1.00
```

NB-I 181 isolated

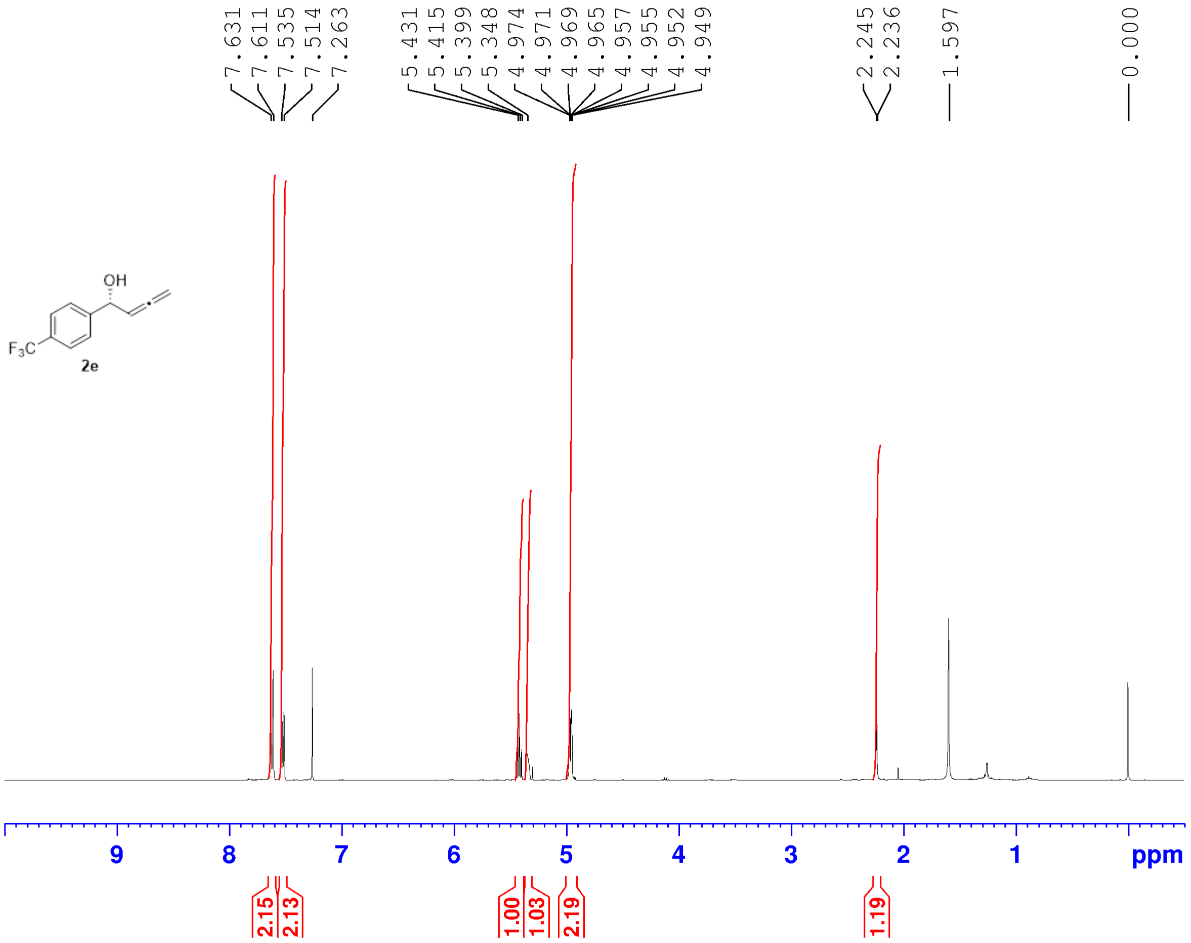

2e

Current Data Parameters
NAME May11-2024
EXPNO 1
PROCNO 1

F2 - Acquisition Parameters
Date_ 20240511
Time 11.50
INSTRUM spect
PROBHD 5 mm TBI 1H/31
PULPROG zg30
TD 65536
SOLVENT CDCl3
NS 16
DS 2
SWH 8278.146 Hz
FIDRES 0.126314 Hz
AQ 3.9583745 sec
RG 574.7
DW 60.400 usec
DE 6.50 usec
TE 300.0 K
D1 1.00000000 sec
TD0 1

======== CHANNEL f1 ========
NUC1 1H
P1 18.75 usec
PL1 0.50 dB
SFO1 400.1324710 MHz

F2 - Processing parameters
SI 32768
SF 400.1300087 MHz
WDW EM
SSB 0
LB 0.30 Hz
GB 0
PC 1.00

NB-I 179

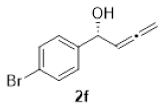

2f

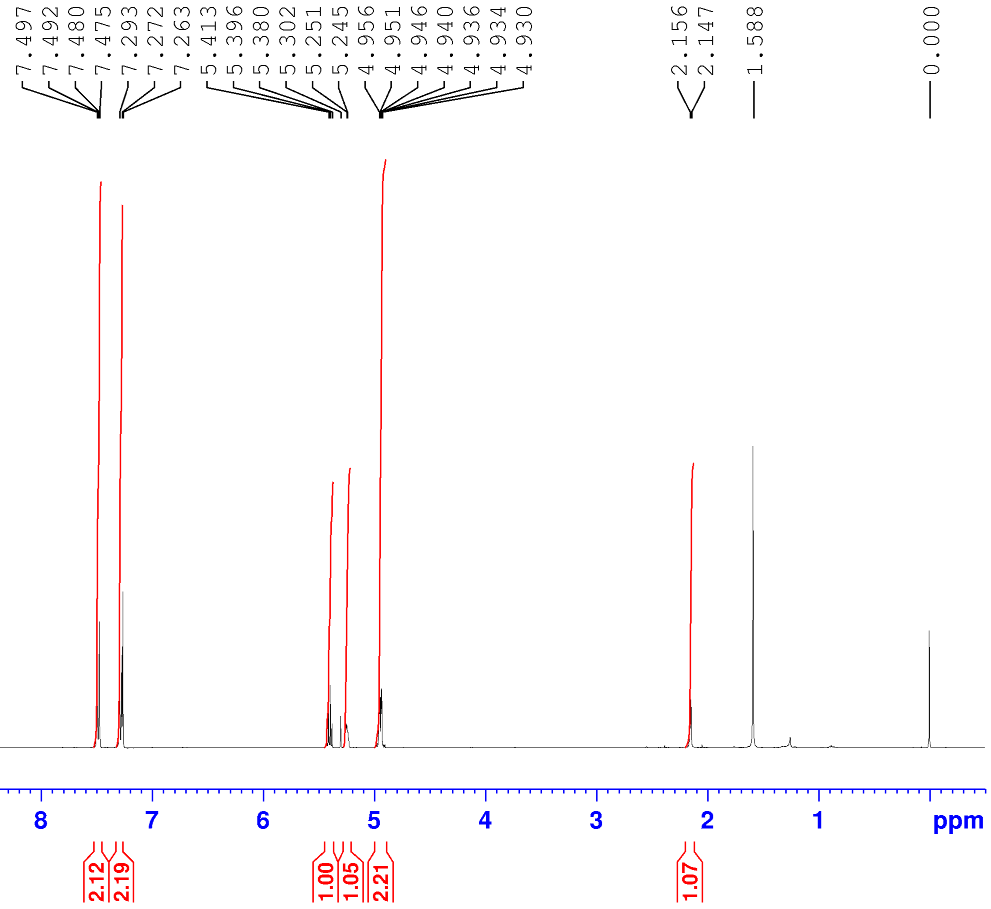

Current Data Parameters	
NAME	May10-2024
EXPNO	3
PROCNO	1
**F2 - Acquisition Parameters**	
Date_	20240510
Time	15.27
INSTRUM	spect
PROBHD	5 mm TBI 1H/31
PULPROG	zg30
TD	65536
SOLVENT	CDCl3
NS	16
DS	2
SWH	8278.146 Hz
FIDRES	0.126314 Hz
AQ	3.9583745 sec
RG	645.1
DW	60.400 usec
DE	6.50 usec
TE	300.0 K
D1	1.00000000 sec
TD0	1
**======== CHANNEL f1 ========**	
NUC1	1H
P1	18.75 usec
PL1	0.50 dB
SFO1	400.1324710 MHz
**F2 - Processing parameters**	
SI	32768
SF	400.1300085 MHz
WDW	EM
SSB	0
LB	0.30 Hz
GB	0
PC	1.00

NB-I 176 isolated

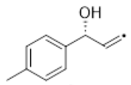

2g

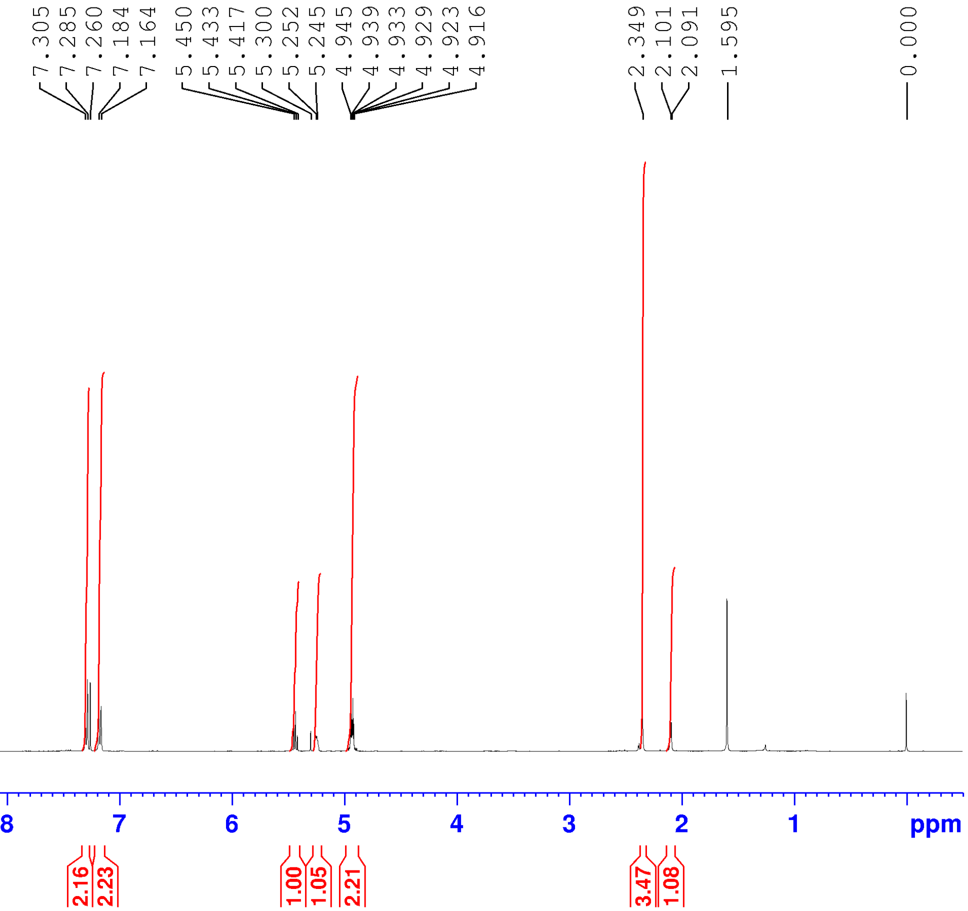

Current Data Parameters	
NAME	May09-2024
EXPNO	2
PROCNO	1
F2 - Acquisition Parameters	
Date_	20240509
Time	12.36
INSTRUM	spect
PROBHD	5 mm TBI 1H/31
PULPROG	zg30
TD	65536
SOLVENT	CDCl3
NS	16
DS	2
SWH	8278.146 Hz
FIDRES	0.126314 Hz
AQ	3.9583745 sec
RG	574.7
DW	60.400 usec
DE	6.50 usec
TE	300.0 K
D1	1.00000000 sec
TD0	1
======== CHANNEL f1 ========	
NUC1	1H
P1	18.75 usec
PL1	0.50 dB
SFO1	400.1324710 MHz
F2 - Processing parameters	
SI	32768
SF	400.1300095 MHz
WDW	EM
SSB	0
LB	0.30 Hz
GB	0
PC	1.00

NB-I 173 isolated

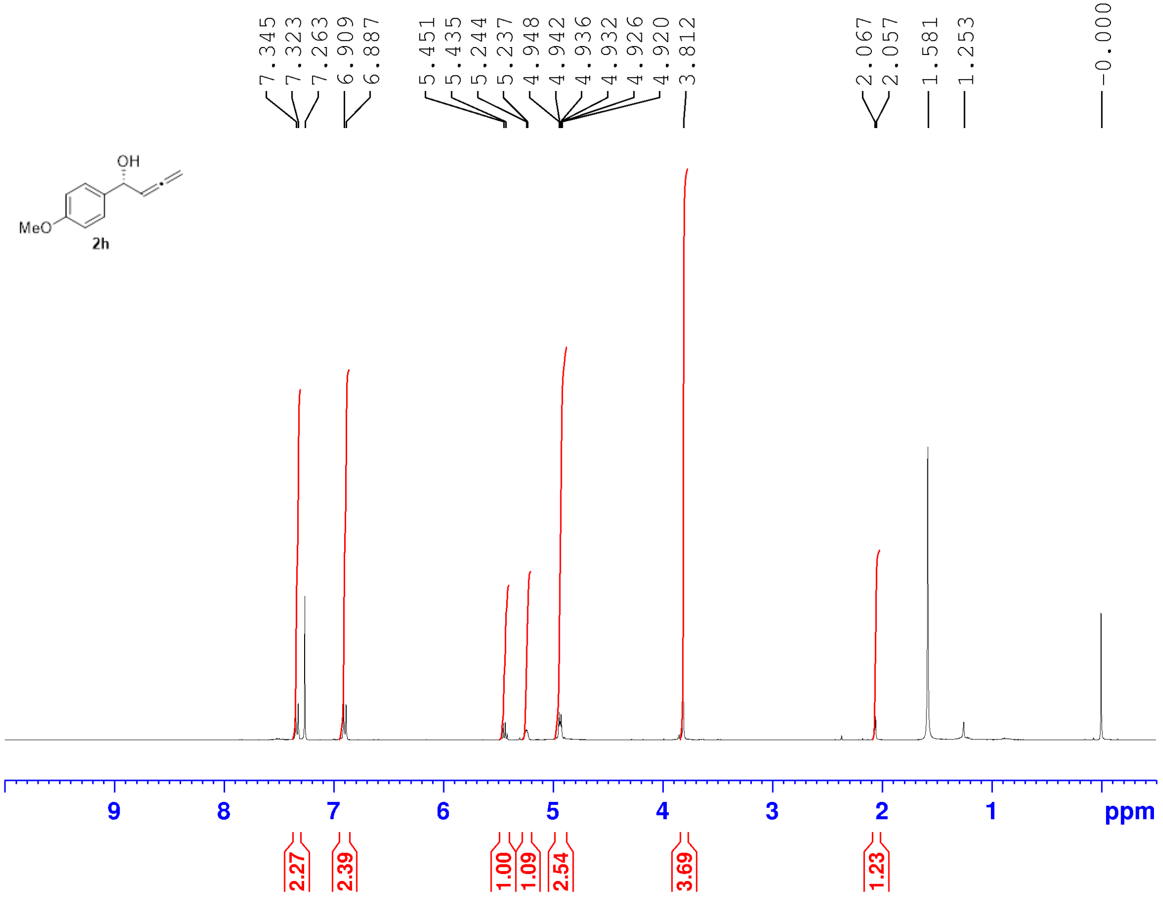

Current Data Parameters
NAME May09-2024
EXPNO 1
PROCNO 1

F2 - Acquisition Parameters
Date_ 20240509
Time 9.56
INSTRUM spect
PROBHD 5 mm TBI 1H/31
PULPROG zg30
TD 65536
SOLVENT CDCl3
NS 16
DS 2
SWH 8278.146 Hz
FIDRES 0.126314 Hz
AQ 3.9583745 sec
RG 1024
DW 60.400 usec
DE 6.50 usec
TE 300.0 K
D1 1.00000000 sec
TD0 1

======== CHANNEL f1 ========
NUC1 1H
P1 18.75 usec
PL1 0.50 dB
SFO1 400.1324710 MHz

F2 - Processing parameters
SI 32768
SF 400.1300085 MHz
WDW EM
SSB 0
LB 0.30 Hz
GB 0
PC 1.00

NB-I 178 isolated

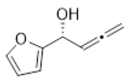

2i

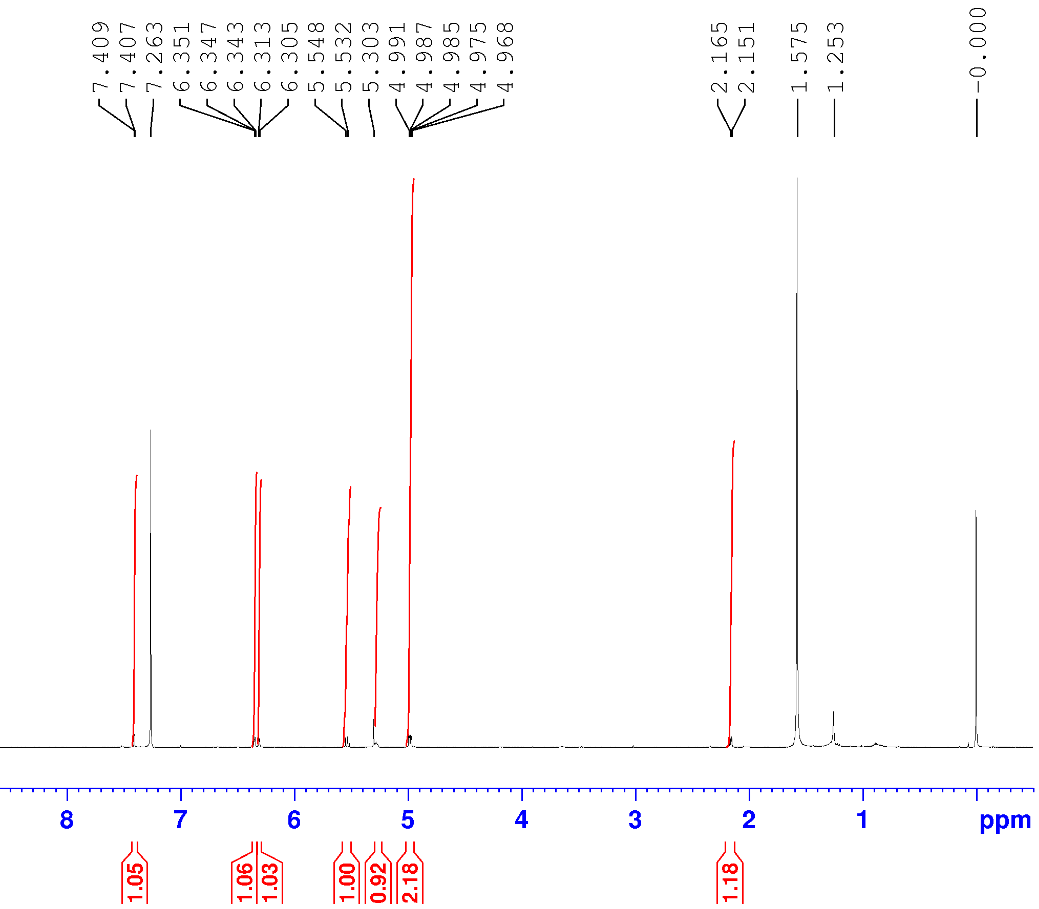


```
Current Data Parameters
NAME          May11-2024
EXPNO                  2
PROCNO                 1

F2 - Acquisition Parameters
Date_           20240511
Time               14.07
INSTRUM            spect
PROBHD   5 mm TBI 1H/31
PULPROG             zg30
TD                 65536
SOLVENT            CDCl3
NS                    16
DS                     2
SWH             8278.146 Hz
FIDRES          0.126314 Hz
AQ             3.9583745 sec
RG                1290.2
DW                60.400 usec
DE                  6.50 usec
TE                 300.0 K
D1            1.00000000 sec
TD0                    1

======== CHANNEL f1 ========
NUC1                  1H
P1                 18.75 usec
PL1                 0.50 dB
SFO1         400.1324710 MHz

F2 - Processing parameters
SI                 32768
SF           400.1300085 MHz
WDW                   EM
SSB                    0
LB                  0.30 Hz
GB                     0
PC                  1.00
```

NB-I 177 isolated

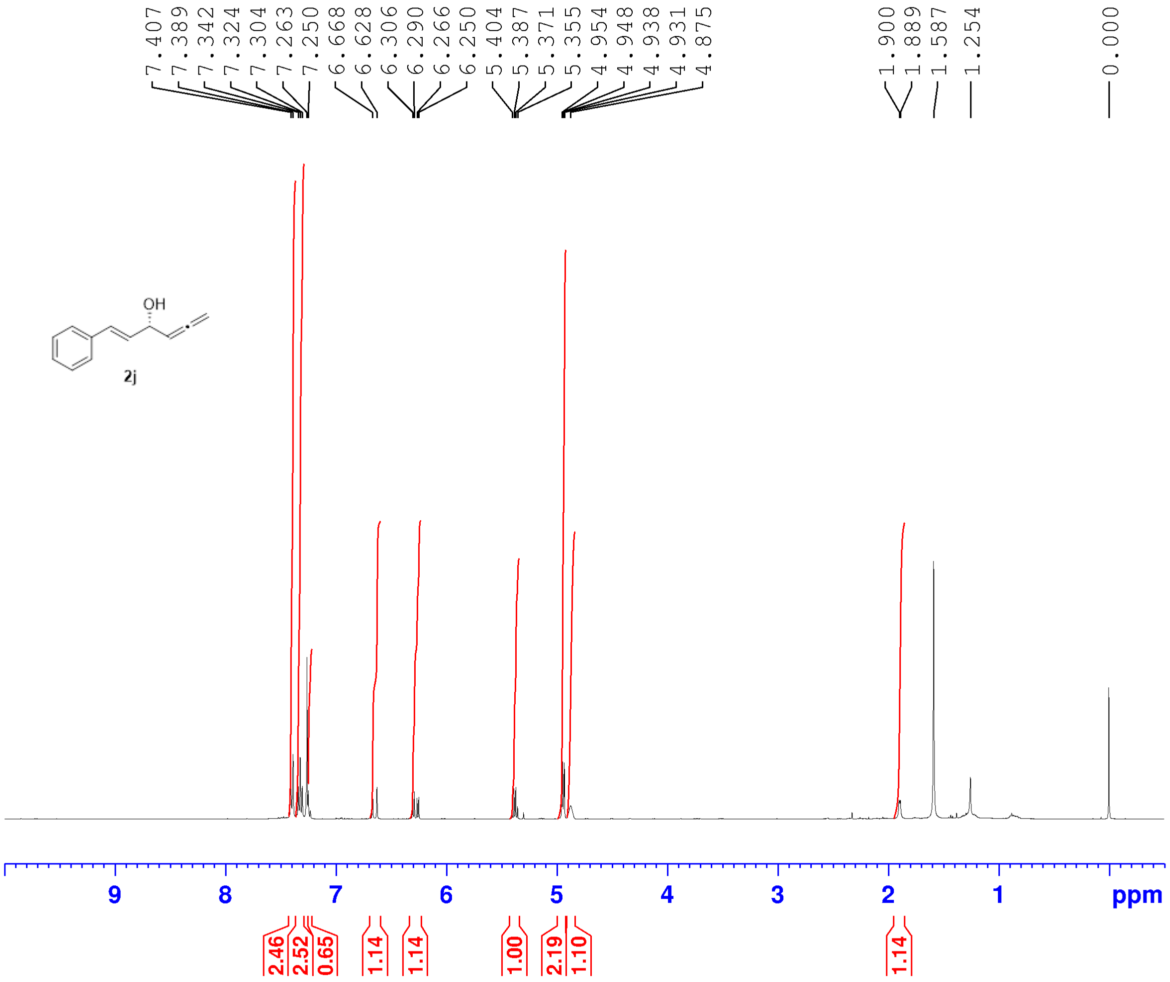

2j


```
Current Data Parameters
NAME             May09-2024
EXPNO                     3
PROCNO                    1

F2 - Acquisition Parameters
Date_              20240509
Time                  15.52
INSTRUM               spect
PROBHD   5 mm TBI 1H/31
PULPROG                zg30
TD                    65536
SOLVENT               CDCl3
NS                       16
DS                        2
SWH                8278.146 Hz
FIDRES             0.126314 Hz
AQ                3.9583745 sec
RG                    812.7
DW                   60.400 usec
DE                     6.50 usec
TE                    300.0 K
D1               1.00000000 sec
TD0                       1

======== CHANNEL f1 ========
NUC1                     1H
P1                    18.75 usec
PL1                    0.50 dB
SFO1            400.1324710 MHz

F2 - Processing parameters
SI                    32768
SF              400.1300087 MHz
WDW                      EM
SSB                       0
LB                     0.30 Hz
GB                        0
PC                     1.00
```

NB-I 175 isolated

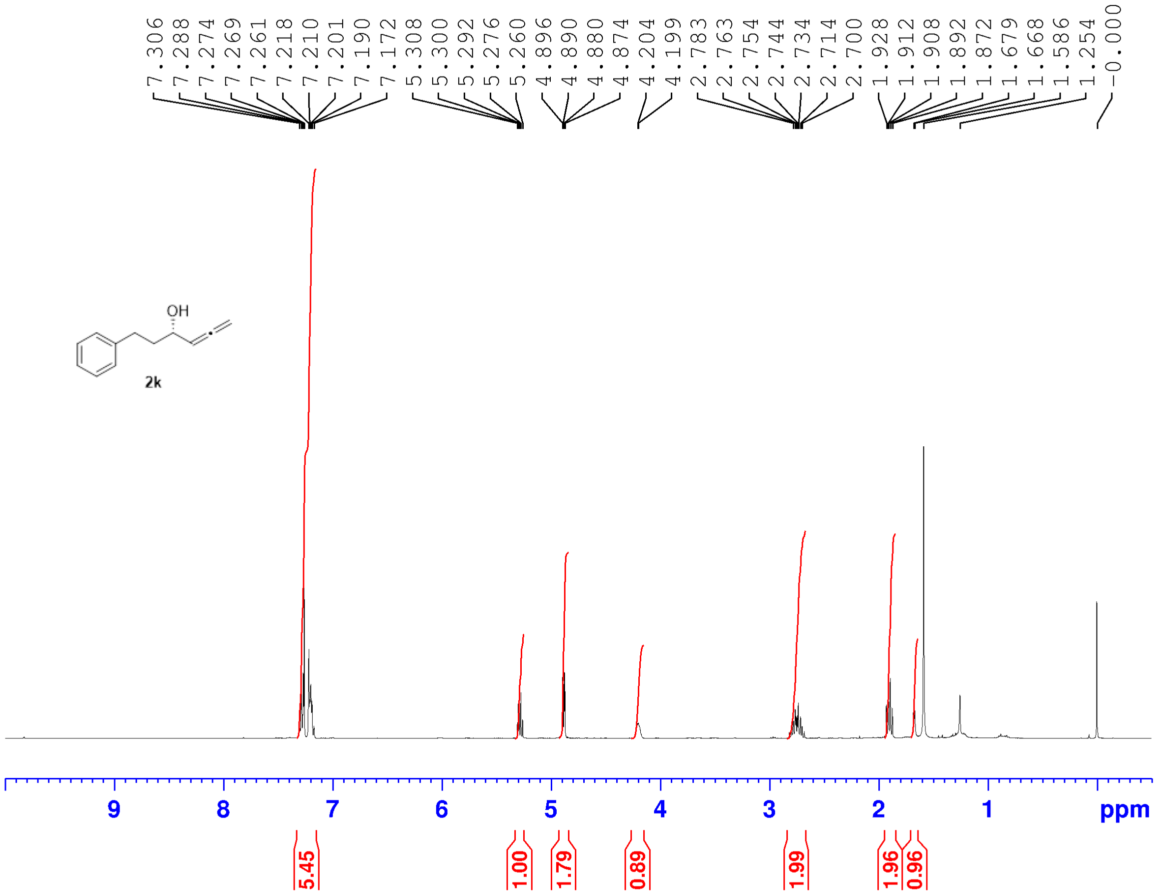


```
Current Data Parameters
NAME              May08-2024
EXPNO                      4
PROCNO                     1

F2 - Acquisition Parameters
Date_               20240508
Time                   20.51
INSTRUM                spect
PROBHD     5 mm TBI 1H/31
PULPROG                 zg30
TD                     65536
SOLVENT                CDCl3
NS                        16
DS                         2
SWH                 8278.146 Hz
FIDRES              0.126314 Hz
AQ                 3.9583745 sec
RG                     574.7
DW                    60.400 usec
DE                      6.50 usec
TE                     300.0 K
D1                1.00000000 sec
TD0                        1

======== CHANNEL f1 ========
NUC1                      1H
P1                     18.75 usec
PL1                     0.50 dB
SFO1             400.1324710 MHz

F2 - Processing parameters
SI                     32768
SF               400.1300092 MHz
WDW                       EM
SSB                        0
LB                      0.30 Hz
GB                         0
PC                      1.00
```

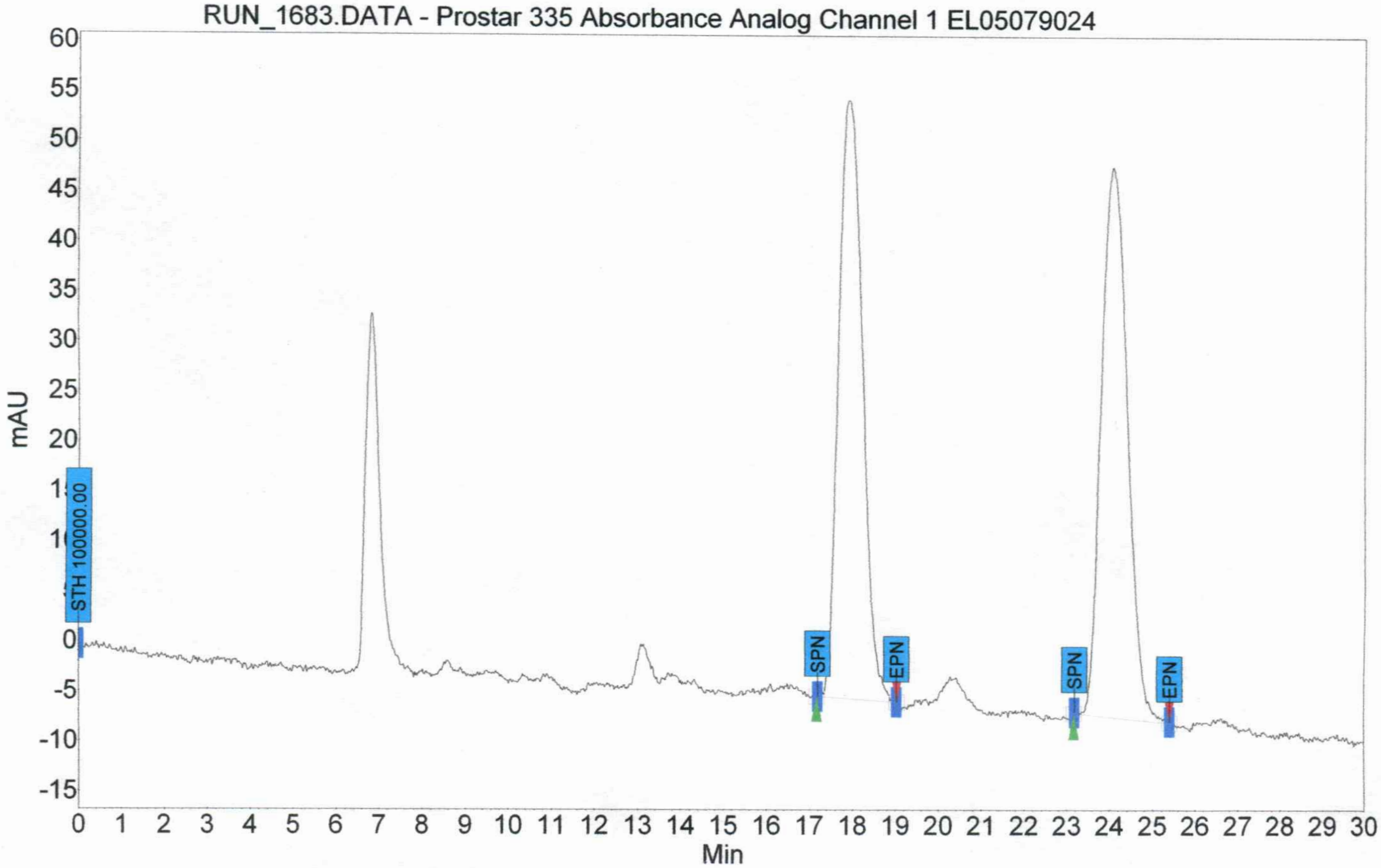

Index	Name	Time [Min]	Quantity [% Area]	Height [mAU]	Area [mAU.Min]	Area % [%]
1	UNKNOWN	17.92	49.68	59.9	38.7	49.681
2	UNKNOWN	24.08	50.32	55.1	39.2	50.319
Total			100.00	115.0	77.9	100.000

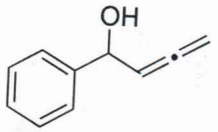

(±)-**2a**

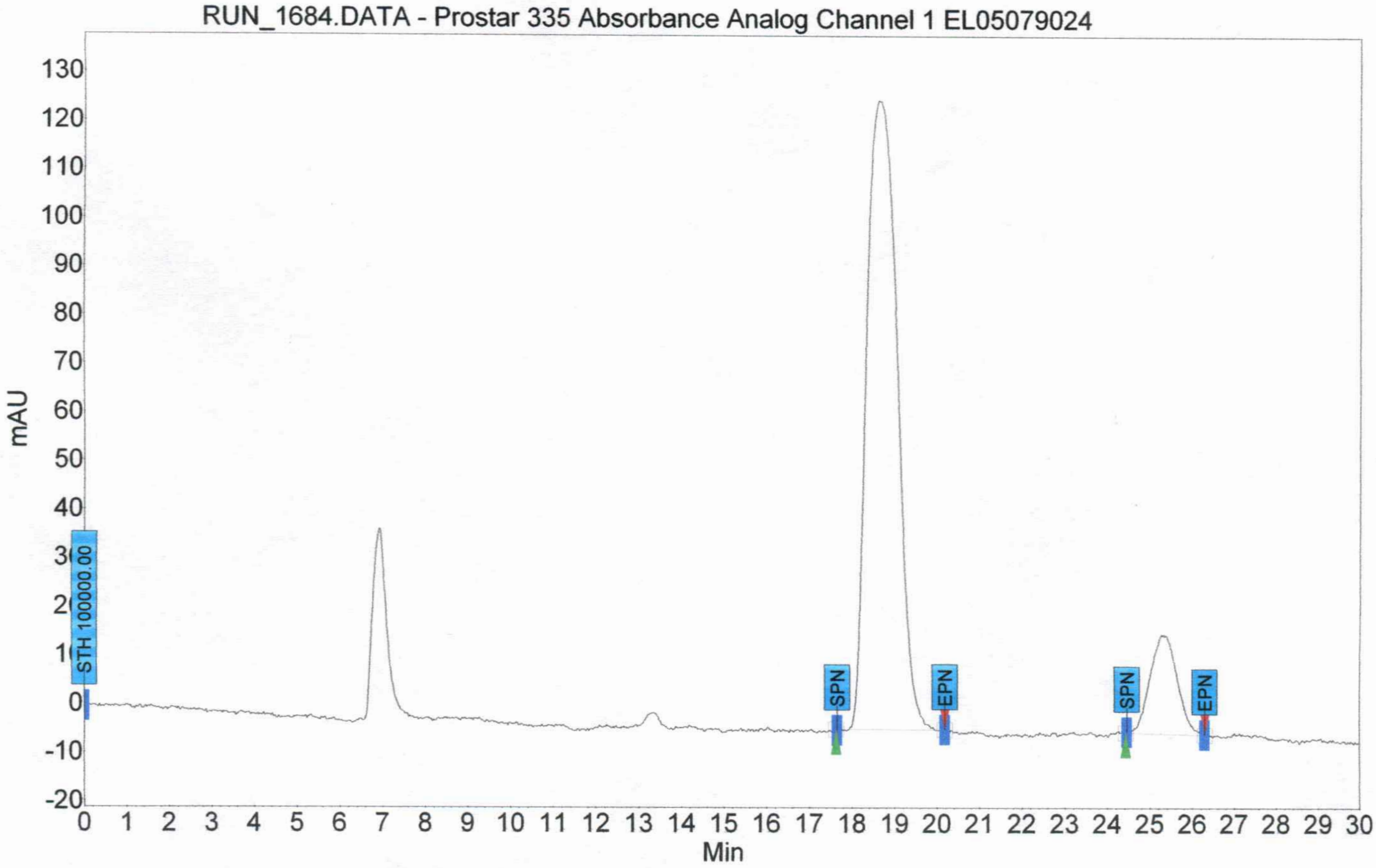

Index	Name	Time [Min]	Quantity [% Area]	Height [mAU]	Area [mAU.Min]	Area % [%]
1	UNKNOWN	18.63	87.57	129.6	106.9	87.570
2	UNKNOWN	25.29	12.43	20.2	15.2	12.430
Total			100.00	149.8	122.1	100.000

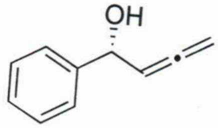

2a

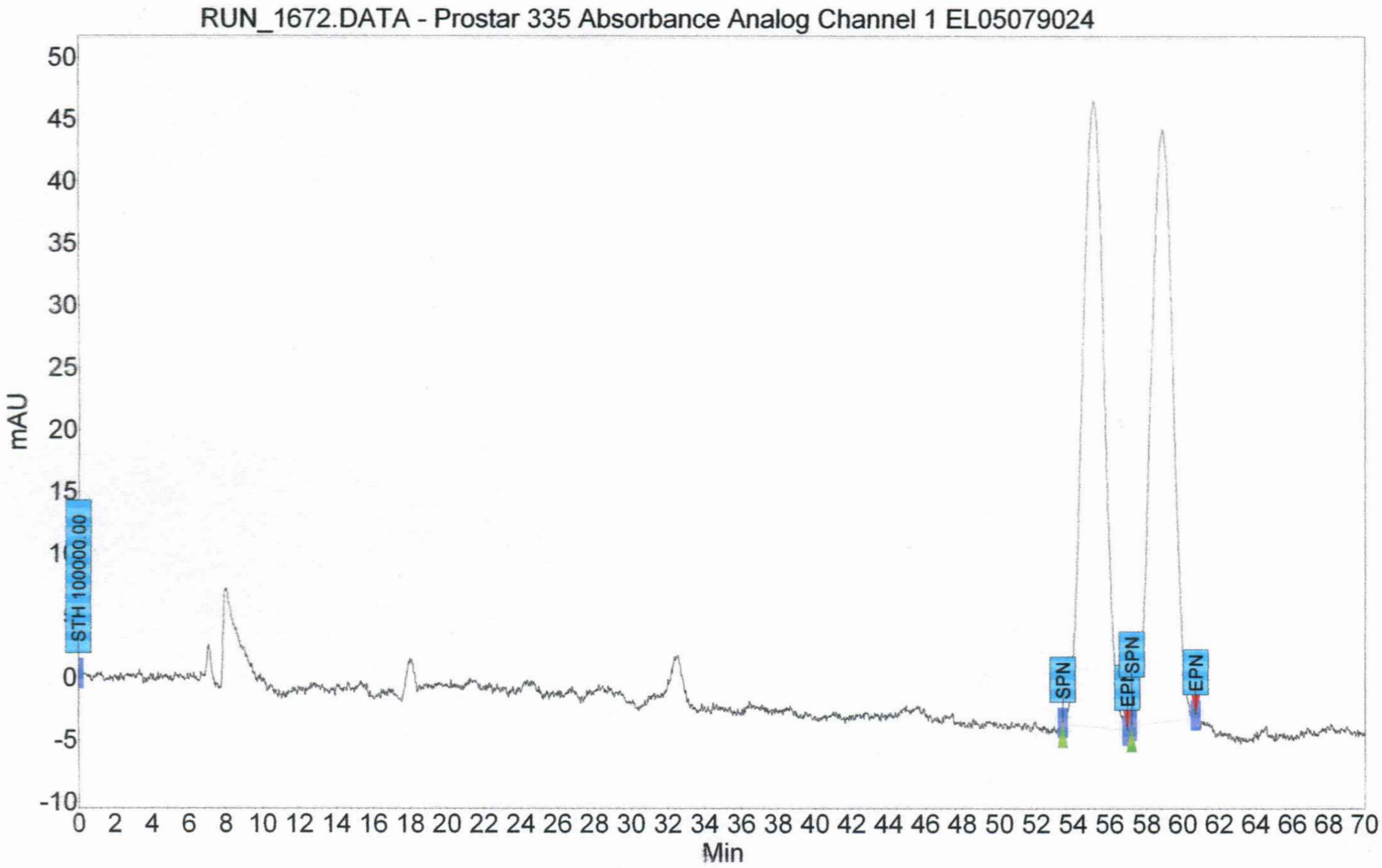

Index	Name	Time [Min]	Quantity [% Area]	Height [mAU]	Area [mAU.Min]	Area % [%]
1	UNKNOWN	55.11	50.80	50.5	68.9	50.799
2	UNKNOWN	58.84	49.20	47.8	66.7	49.201
Total			100.00	98.3	135.6	100.000

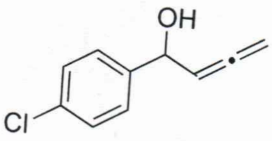

(±)-**2b**

RUN_1674.DATA - Prostar 335 Absorbance Analog Channel 1 EL05079024

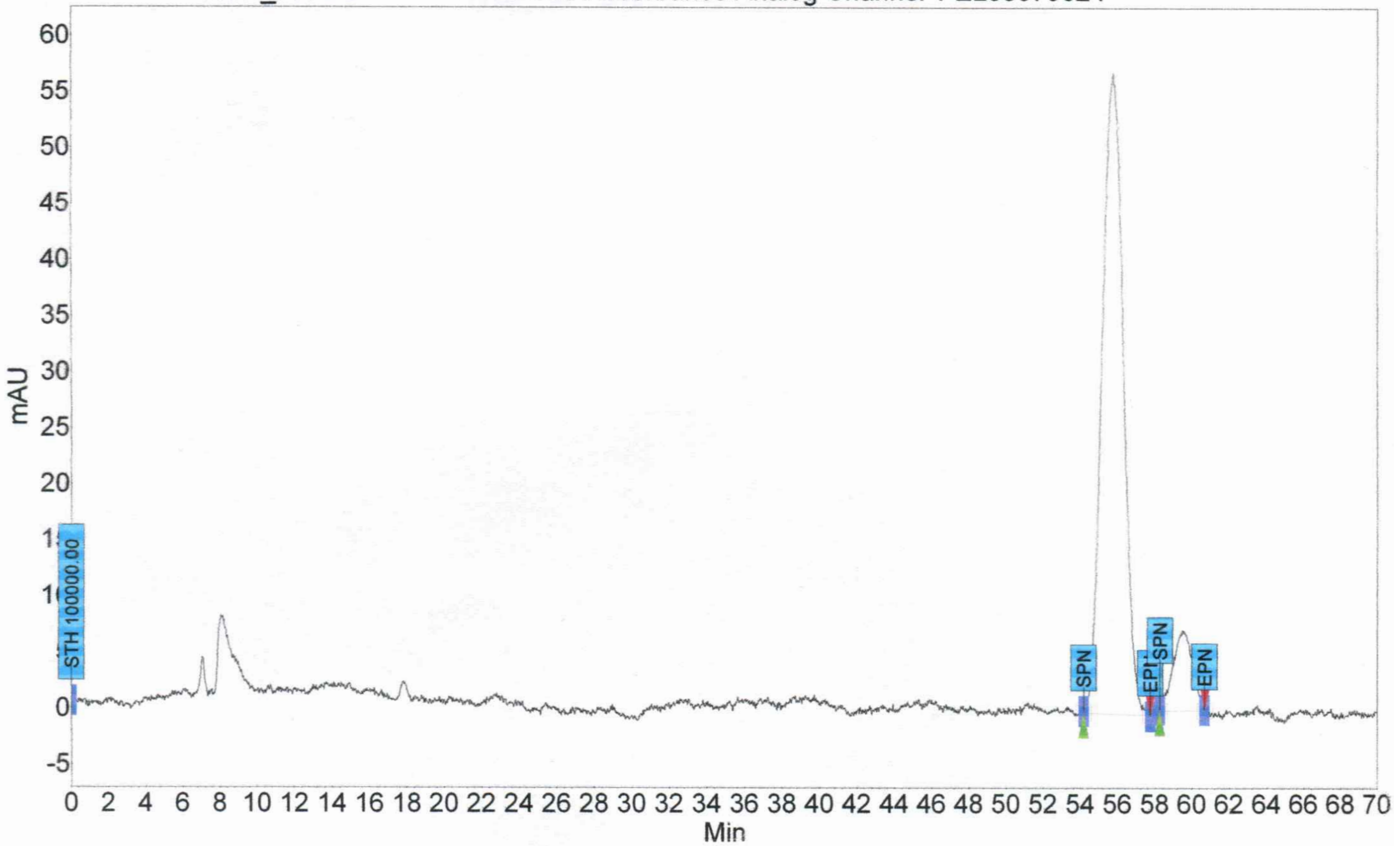

Index	Name	Time [Min]	Quantity [% Area]	Height [mAU]	Area [mAU.Min]	Area % [%]
1	UNKNOWN	55.71	89.85	57.2	74.9	89.852
2	UNKNOWN	59.51	10.15	7.2	8.5	10.148
Total			100.00	64.4	83.3	100.000

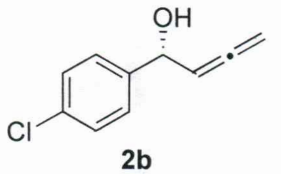

2b

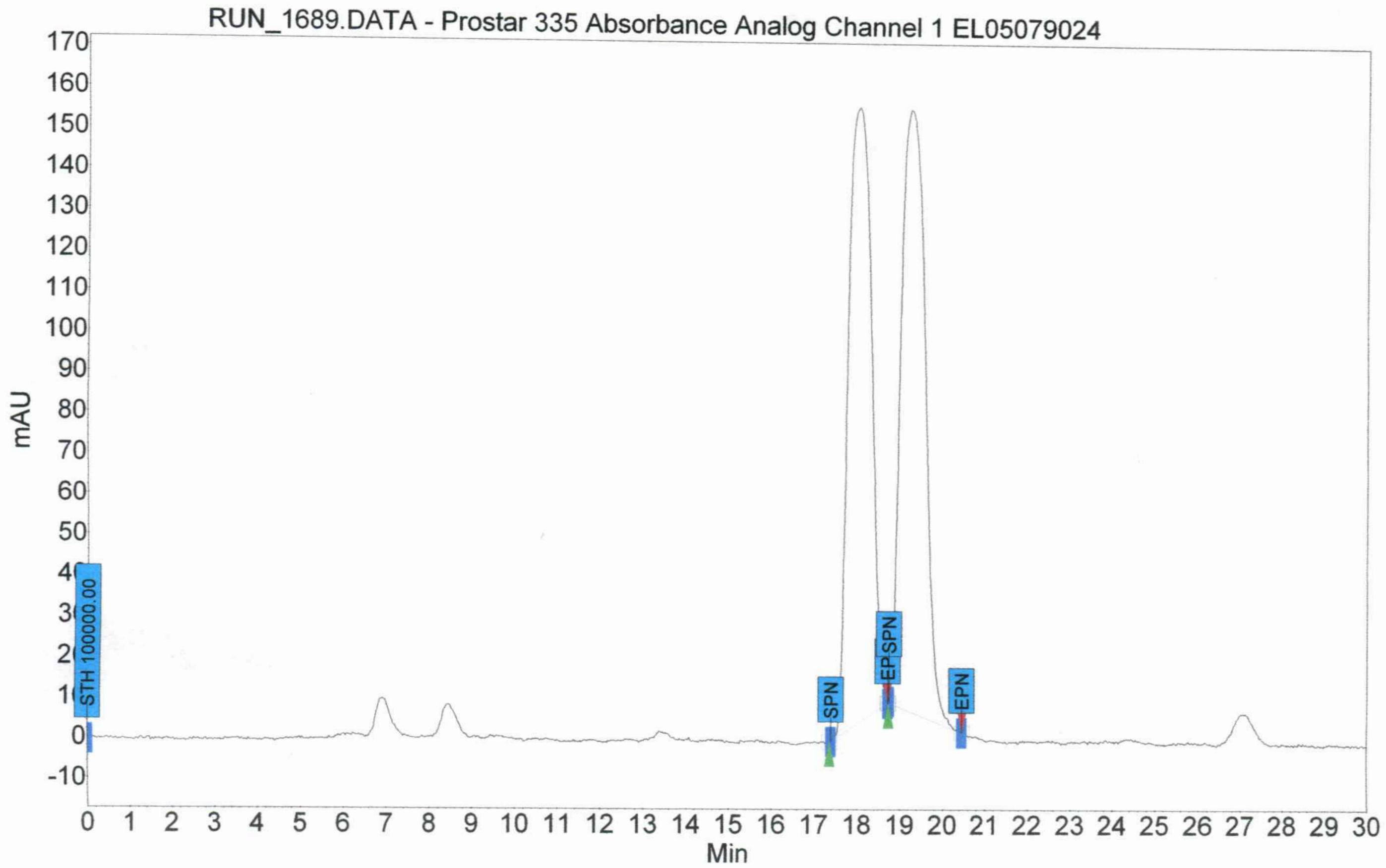

Index	Name	Time [Min]	Quantity [% Area]	Height [mAU]	Area [mAU.Min]	Area % [%]
1	UNKNOWN	18.01	49.44	152.3	95.0	49.437
2	UNKNOWN	19.23	50.56	148.8	97.2	50.563
Total			100.00	301.1	192.2	100.000

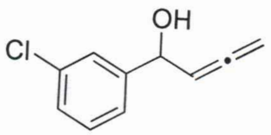

(±)-**2c**

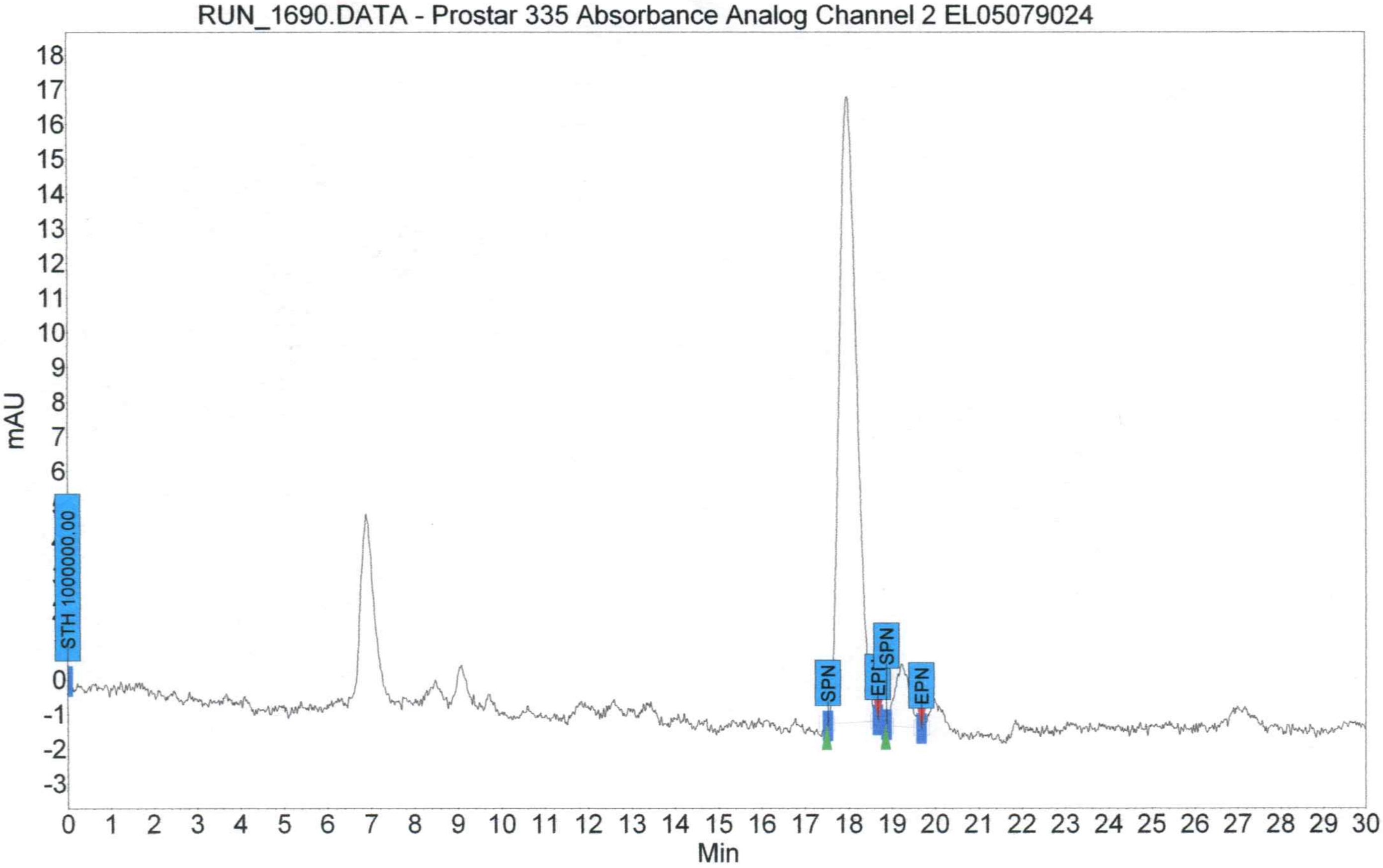

Index	Name	Time [Min]	Quantity [% Area]	Height [mAU]	Area [mAU.Min]	Area % [%]
1	UNKNOWN	17.97	92.40	18.1	8.4	92.402
2	UNKNOWN	19.24	7.60	1.8	0.7	7.598
Total			100.00	19.9	9.1	100.000

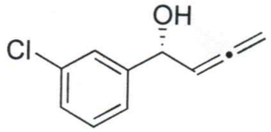

2c

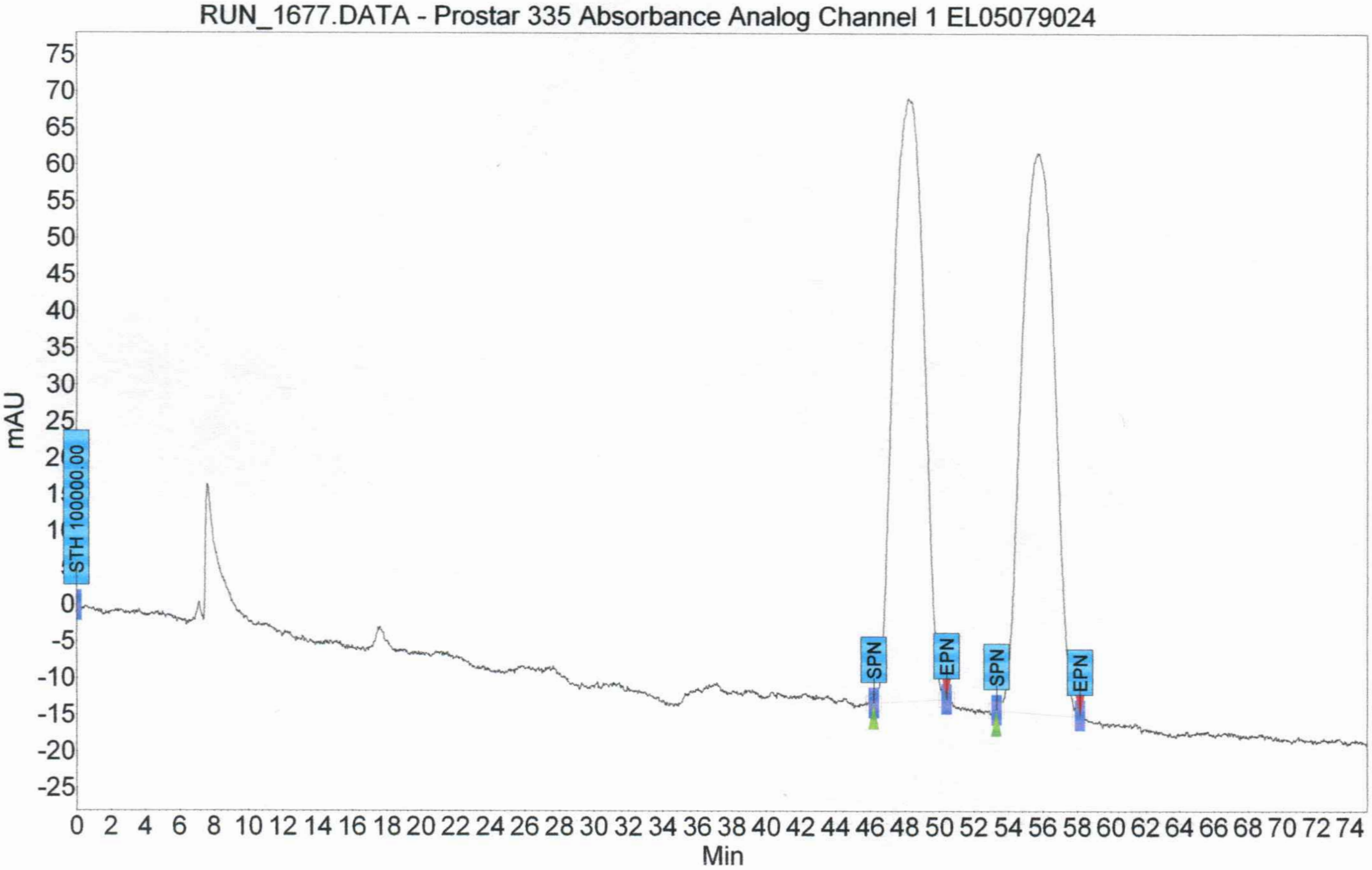

Index	Name	Time [Min]	Quantity [% Area]	Height [mAU]	Area [mAU.Min]	Area % [%]
1	UNKNOWN	48.25	49.68	82.1	167.0	49.681
2	UNKNOWN	55.76	50.32	76.6	169.2	50.319
Total			100.00	158.7	336.2	100.000

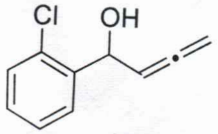

(±)-**2d**

RUN_1678.DATA - Prostar 335 Absorbance Analog Channel 1 EL05079024

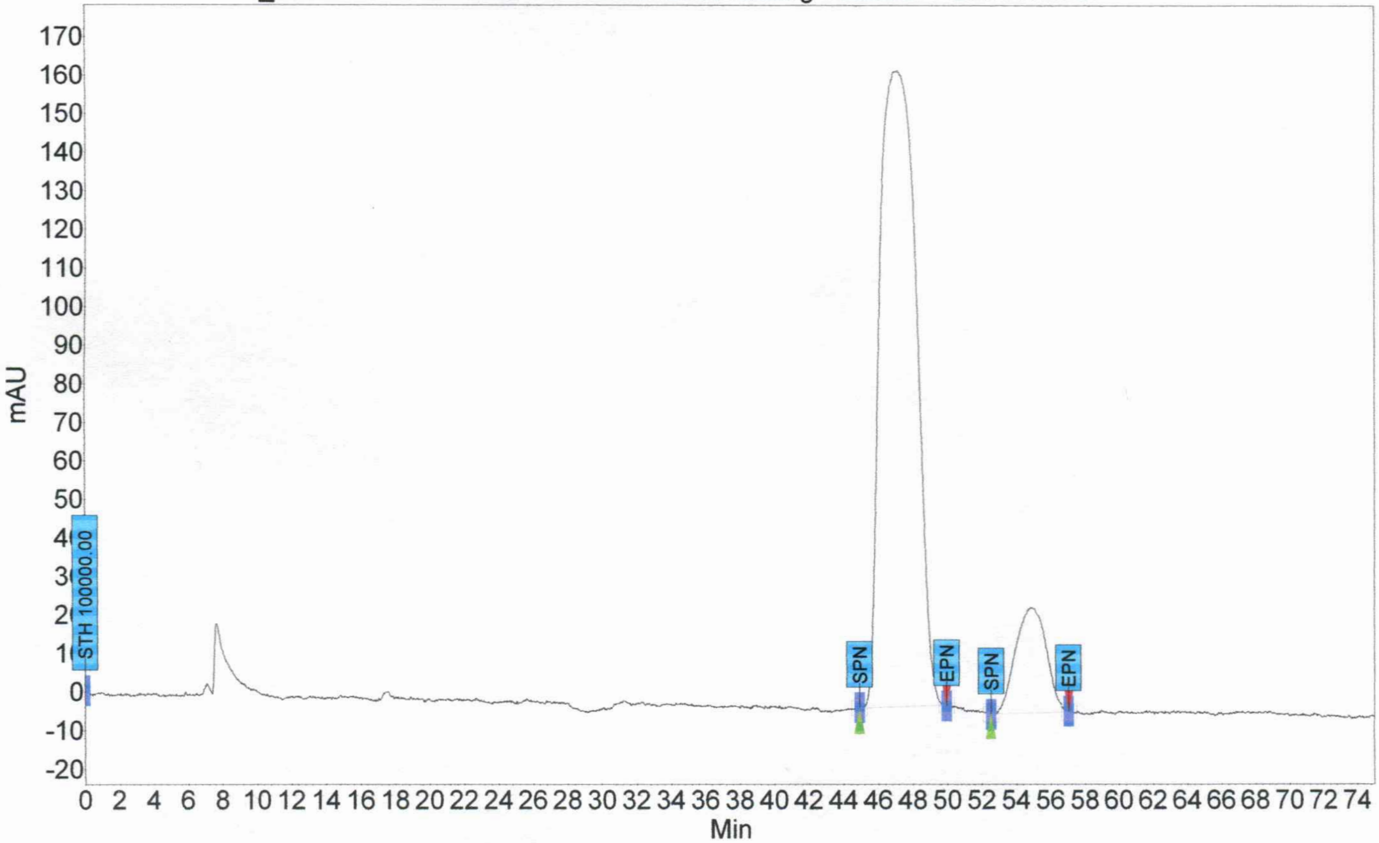

Index	Name	Time [Min]	Quantity [% Area]	Height [mAU]	Area [mAU.Min]	Area % [%]
1	UNKNOWN	47.17	87.56	165.3	400.8	87.560
2	UNKNOWN	54.93	12.44	27.3	56.9	12.440
Total			100.00	192.6	457.8	100.000

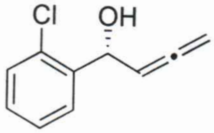

2d

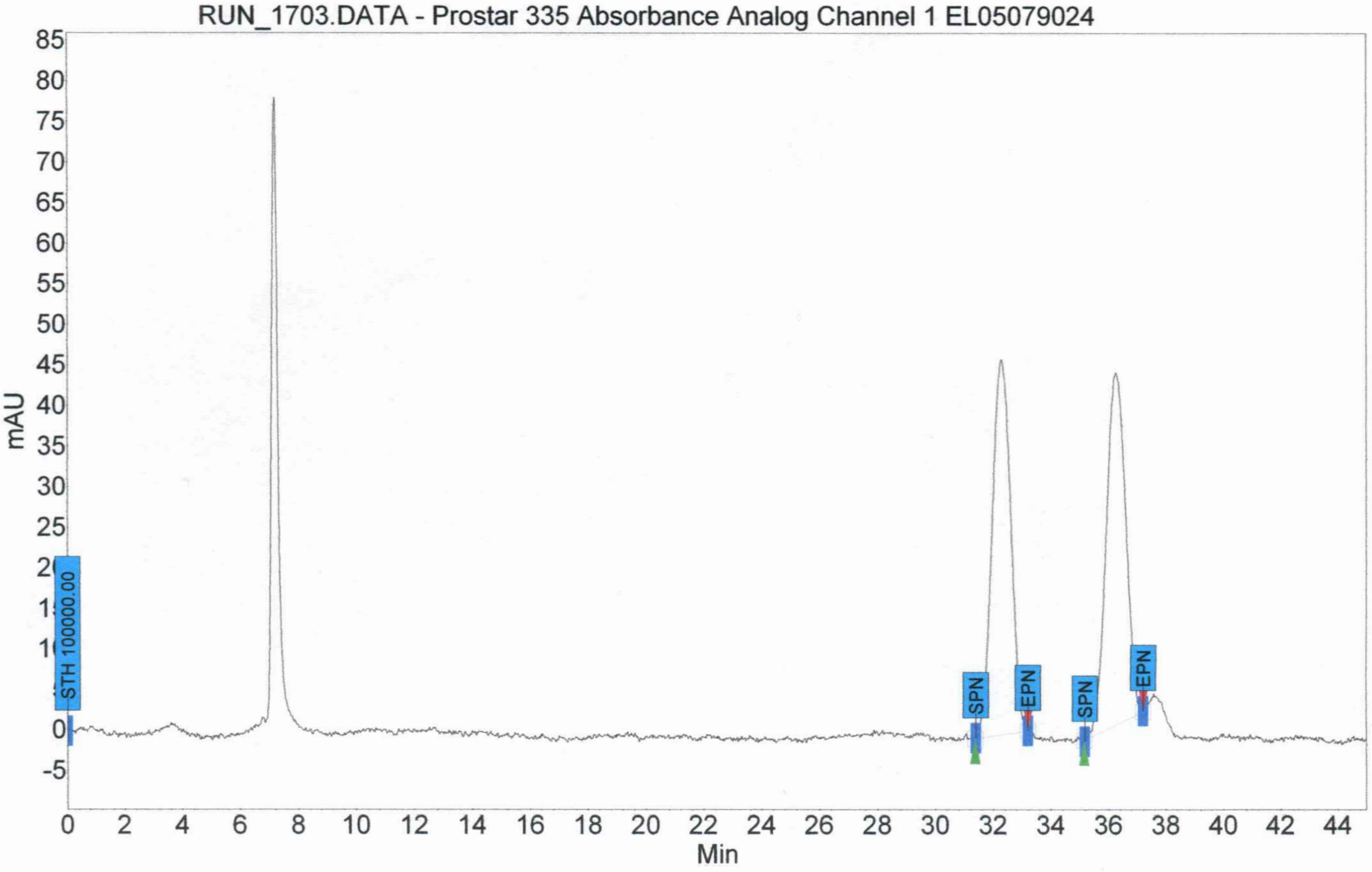

Index	Name	Time [Min]	Quantity [% Area]	Height [mAU]	Area [mAU.Min]	Area % [%]
1	UNKNOWN	32.28	50.24	46.3	34.4	50.237
2	UNKNOWN	36.23	49.76	43.4	34.1	49.763
Total			100.00	89.6	68.4	100.000

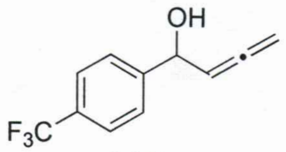

(±)-**2e**

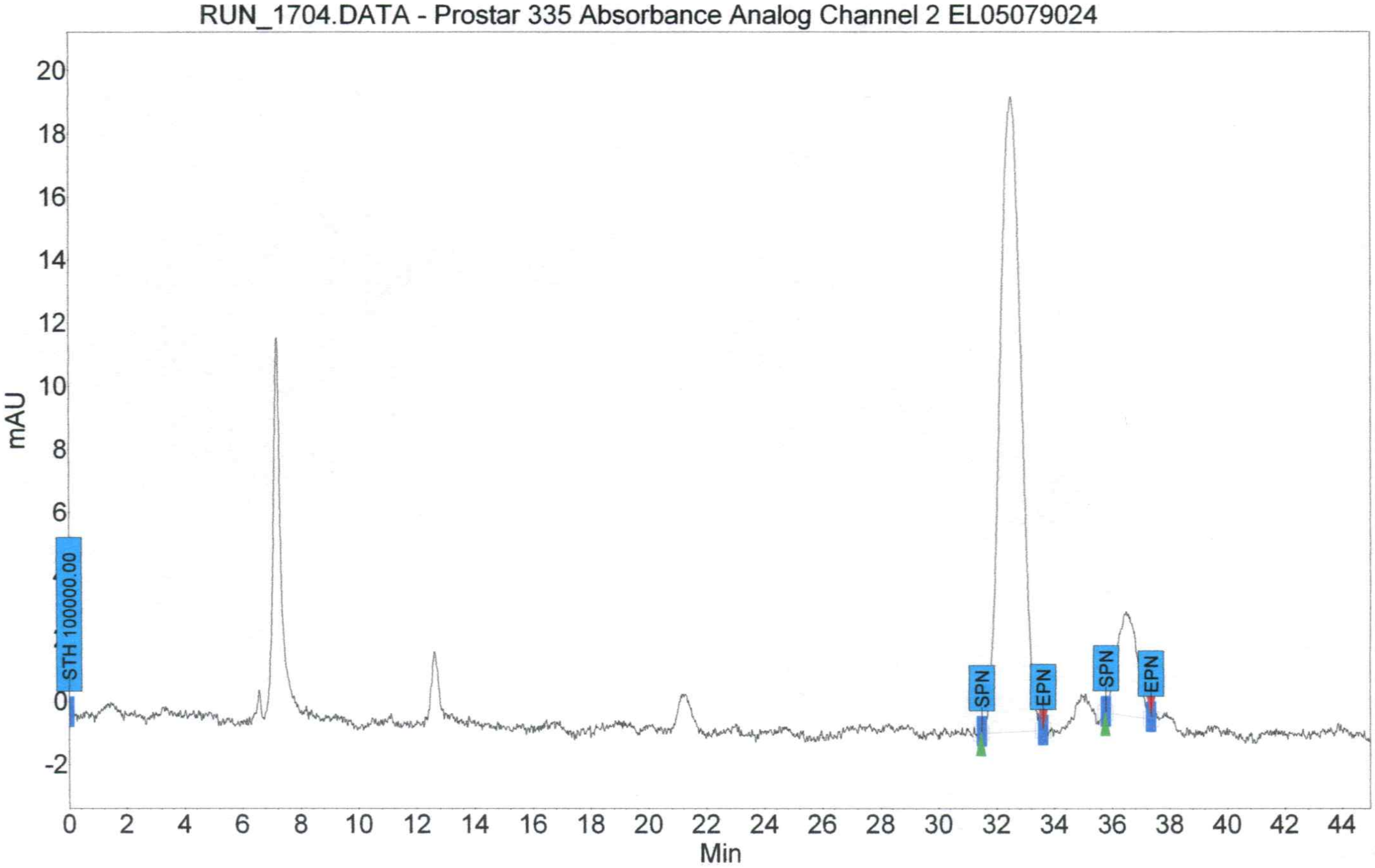

Index	Name	Time [Min]	Quantity [% Area]	Height [mAU]	Area [mAU.Min]	Area % [%]
1	UNKNOWN	32.49	86.54	20.1	16.9	86.540
2	UNKNOWN	36.47	13.46	3.3	2.6	13.460
Total			100.00	23.4	19.6	100.000

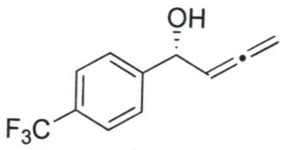

2e

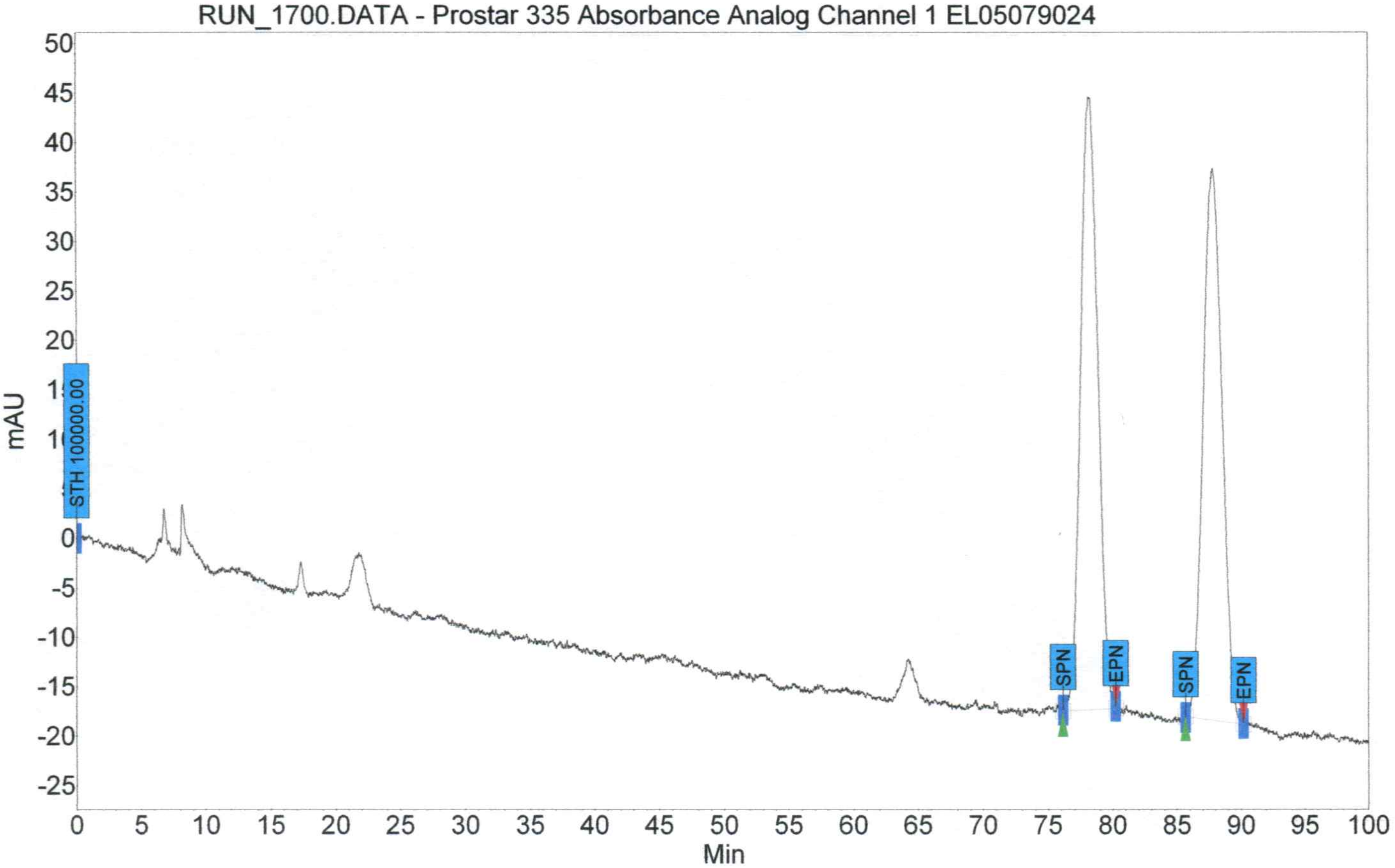

Index	Name	Time [Min]	Quantity [% Area]	Height [mAU]	Area [mAU.Min]	Area % [%]
1	UNKNOWN	78.16	50.15	61.9	96.2	50.155
2	UNKNOWN	87.80	49.85	55.6	95.6	49.845
Total			100.00	117.5	191.9	100.000

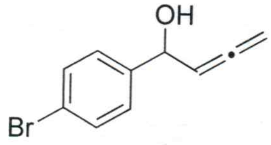

(±)-**2f**

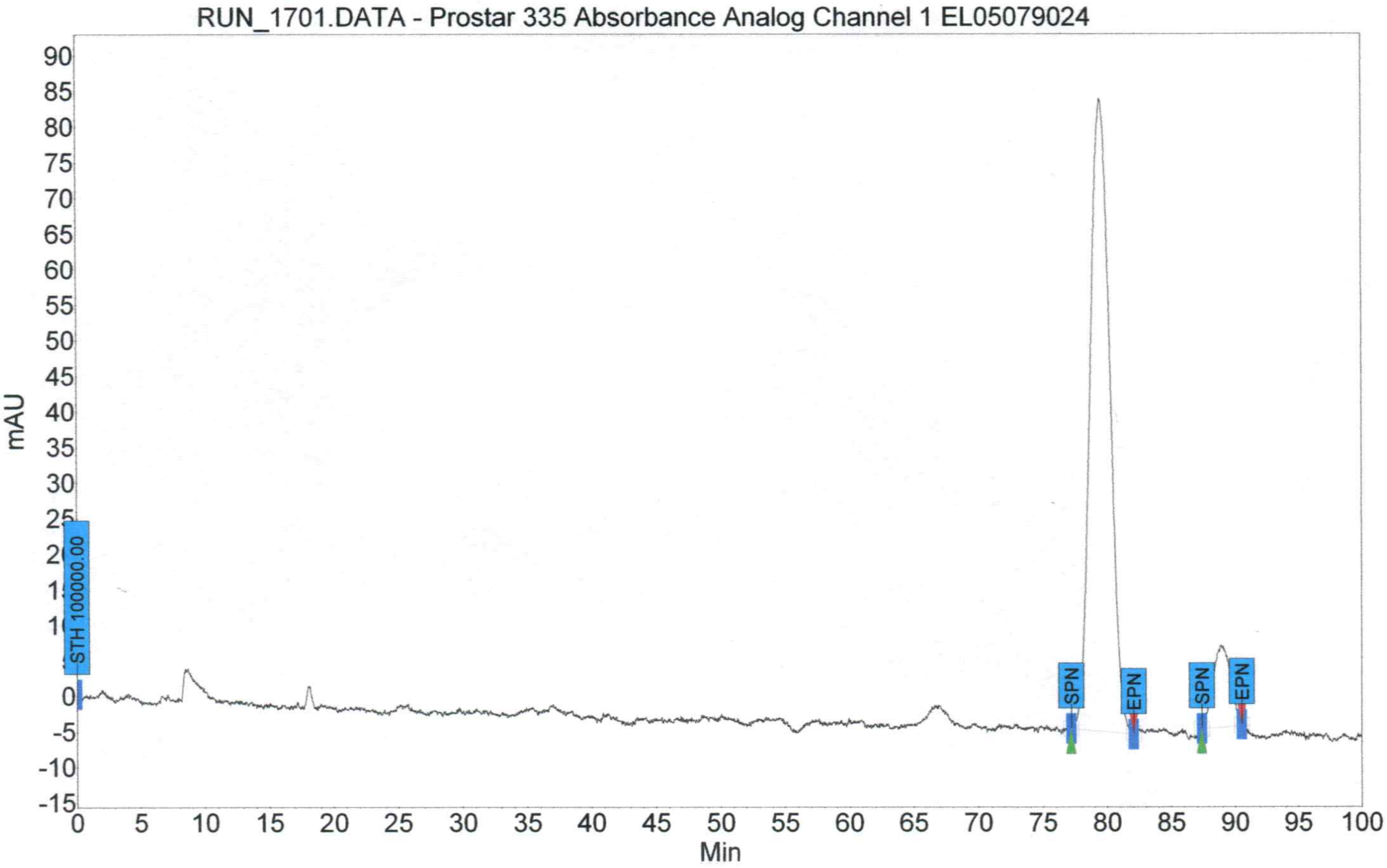

Index	Name	Time [Min]	Quantity [% Area]	Height [mAU]	Area [mAU.Min]	Area % [%]
1	UNKNOWN	79.45	89.49	88.9	157.9	89.492
2	UNKNOWN	88.88	10.51	11.4	18.5	10.508
Total			100.00	100.3	176.5	100.000

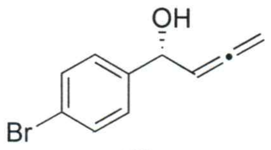

2f

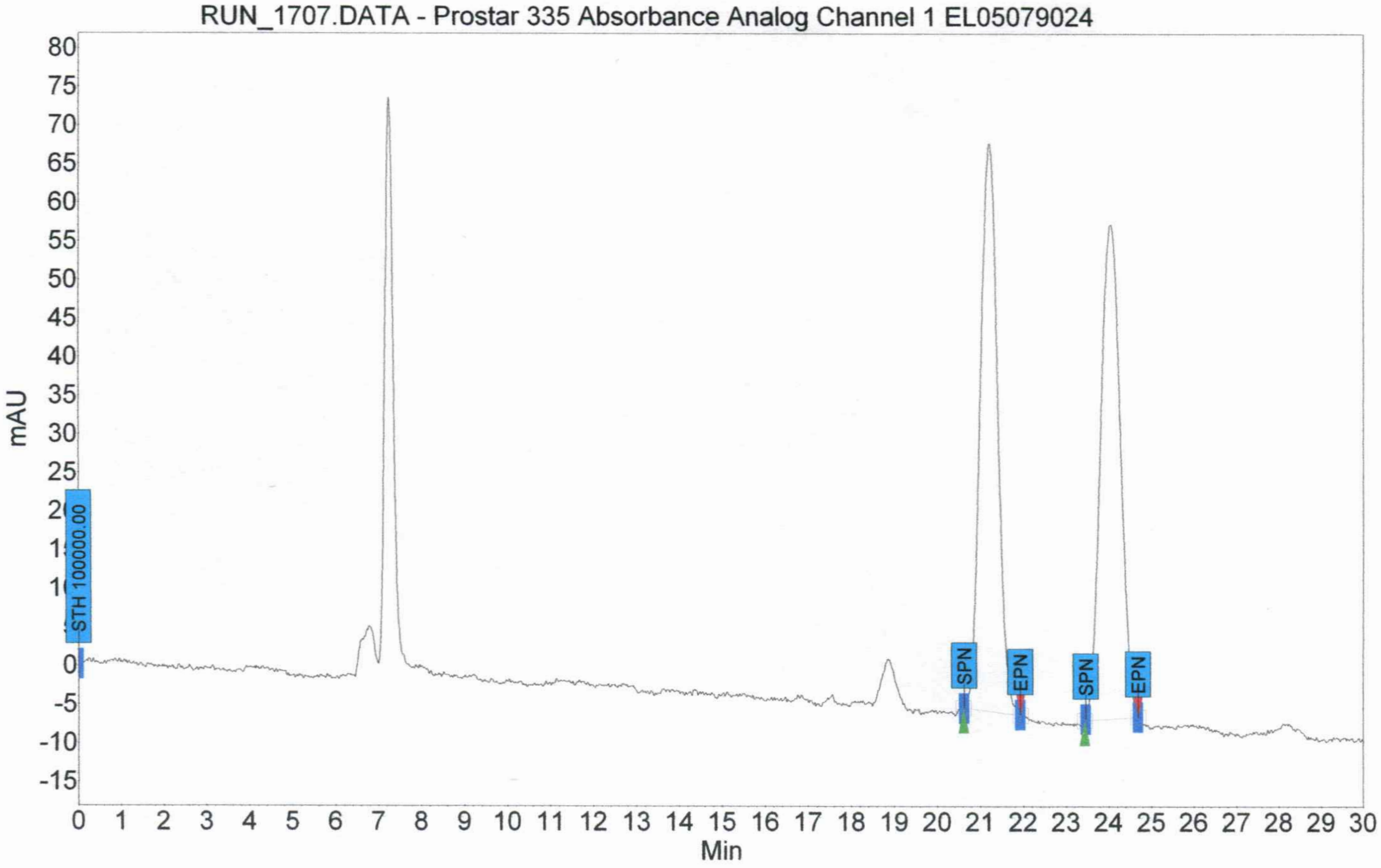

Index	Name	Time [Min]	Quantity [% Area]	Height [mAU]	Area [mAU.Min]	Area % [%]
1	UNKNOWN	21.20	49.89	73.5	34.2	49.888
2	UNKNOWN	24.03	50.11	64.0	34.3	50.112
Total			100.00	137.5	68.5	100.000

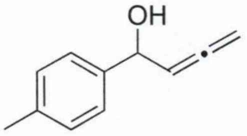

(±)-**2g**

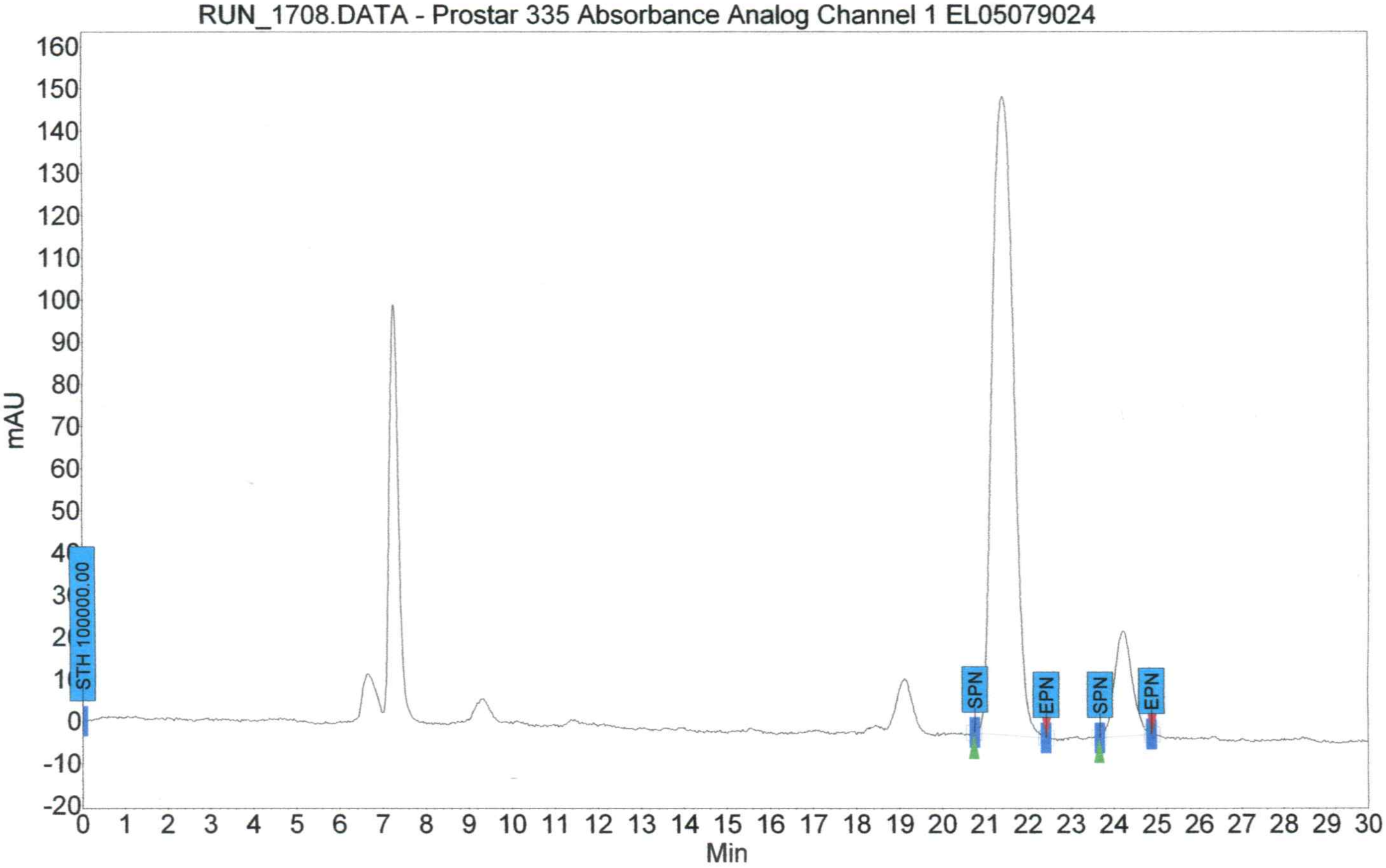

Index	Name	Time [Min]	Quantity [% Area]	Height [mAU]	Area [mAU.Min]	Area % [%]
1	UNKNOWN	21.41	87.94	151.5	87.8	87.937
2	UNKNOWN	24.21	12.06	25.1	12.0	12.063
Total			100.00	176.6	99.8	100.000

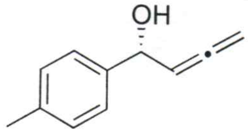

2g

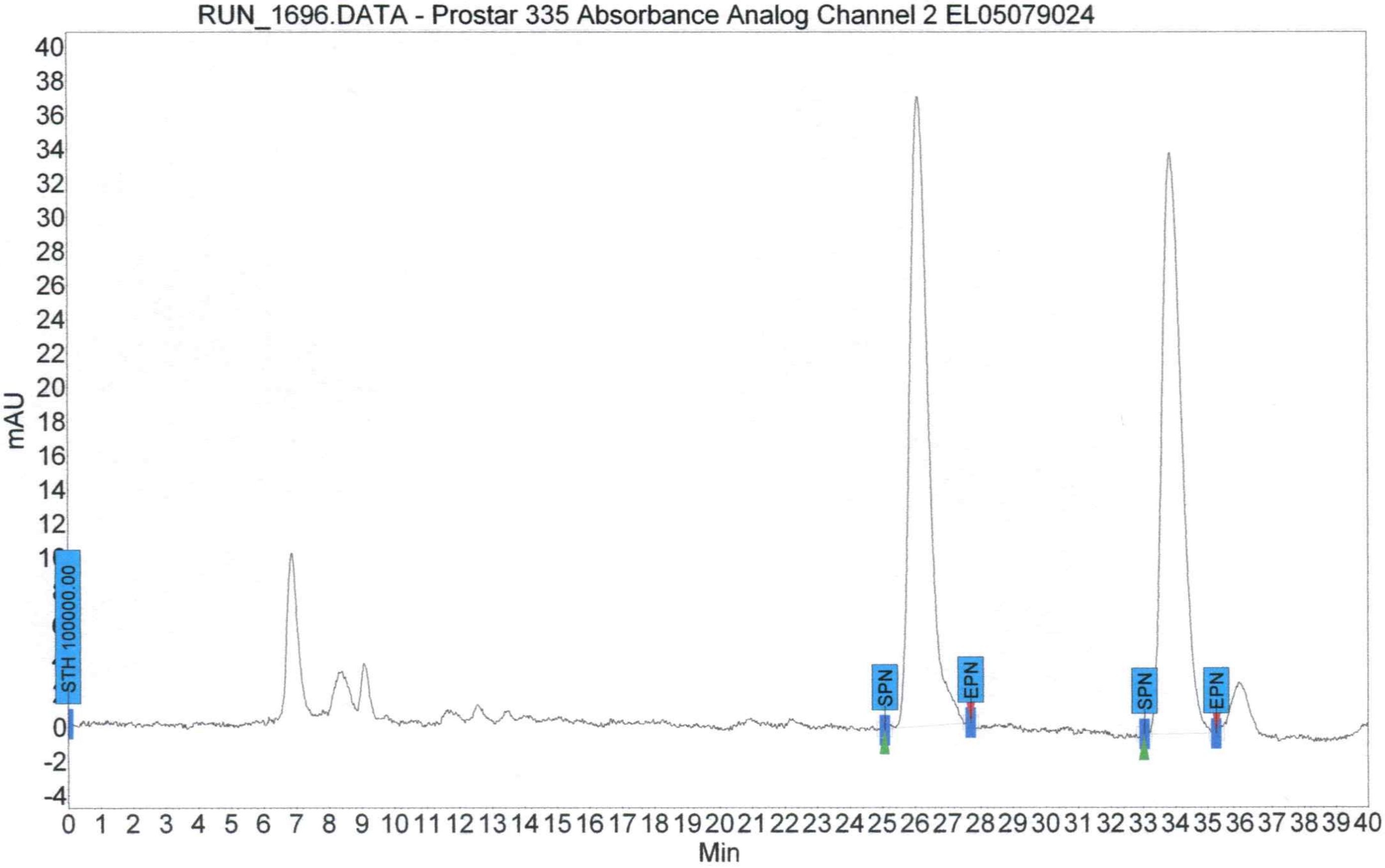

Index	Name	Time [Min]	Quantity [% Area]	Height [mAU]	Area [mAU.Min]	Area % [%]
1	UNKNOWN	26.08	50.32	37.1	24.2	50.322
2	UNKNOWN	33.85	49.68	34.2	23.9	49.678
Total			100.00	71.2	48.2	100.000

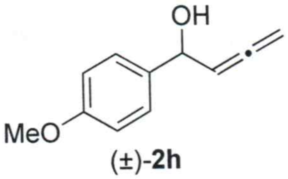

(±)-**2h**

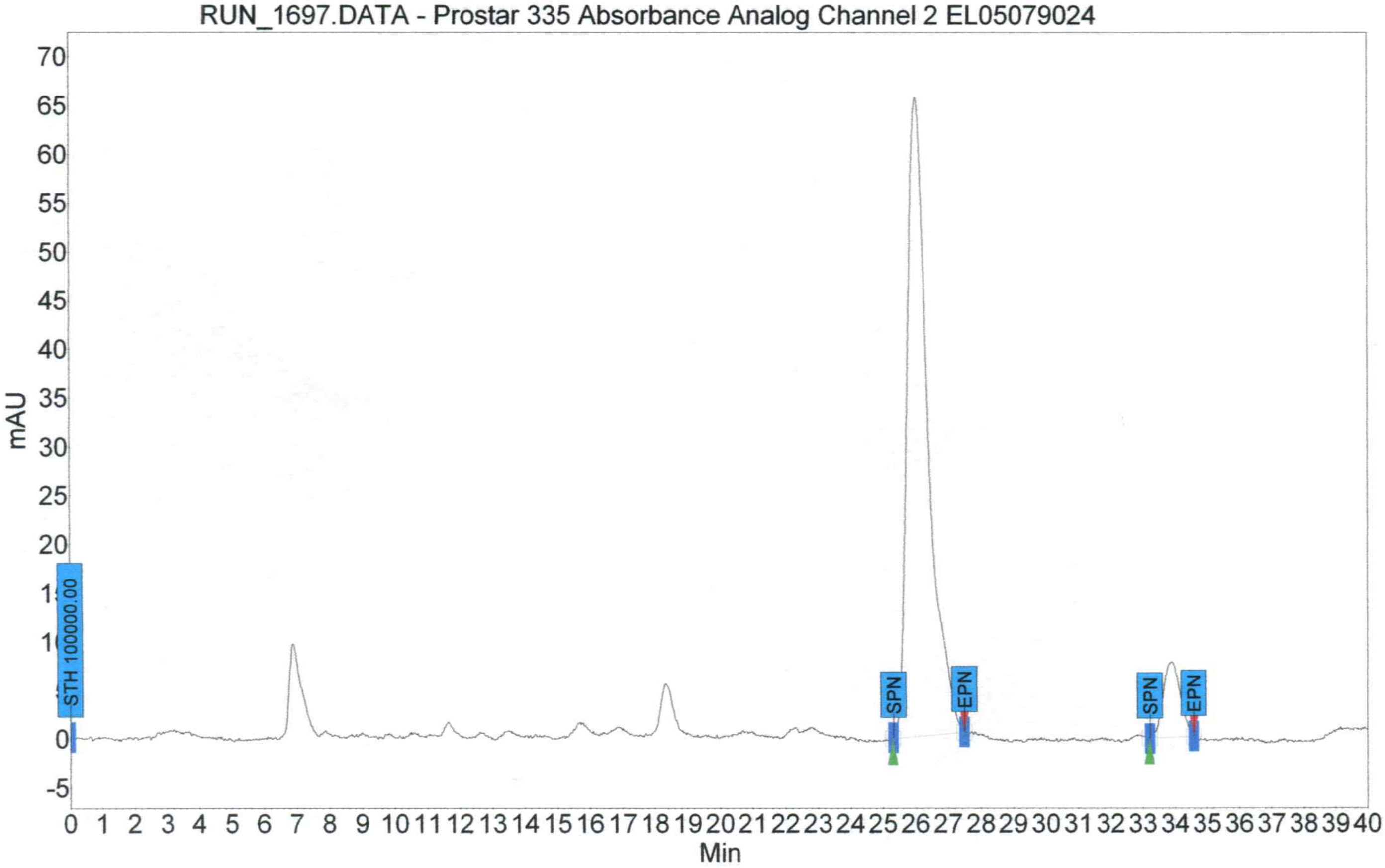

Index	Name	Time [Min]	Quantity [% Area]	Height [mAU]	Area [mAU.Min]	Area % [%]
1	UNKNOWN	26.00	91.09	65.7	49.0	91.093
2	UNKNOWN	33.88	8.91	7.8	4.8	8.907
Total			100.00	73.4	53.7	100.000

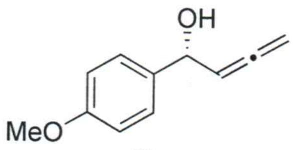

2h

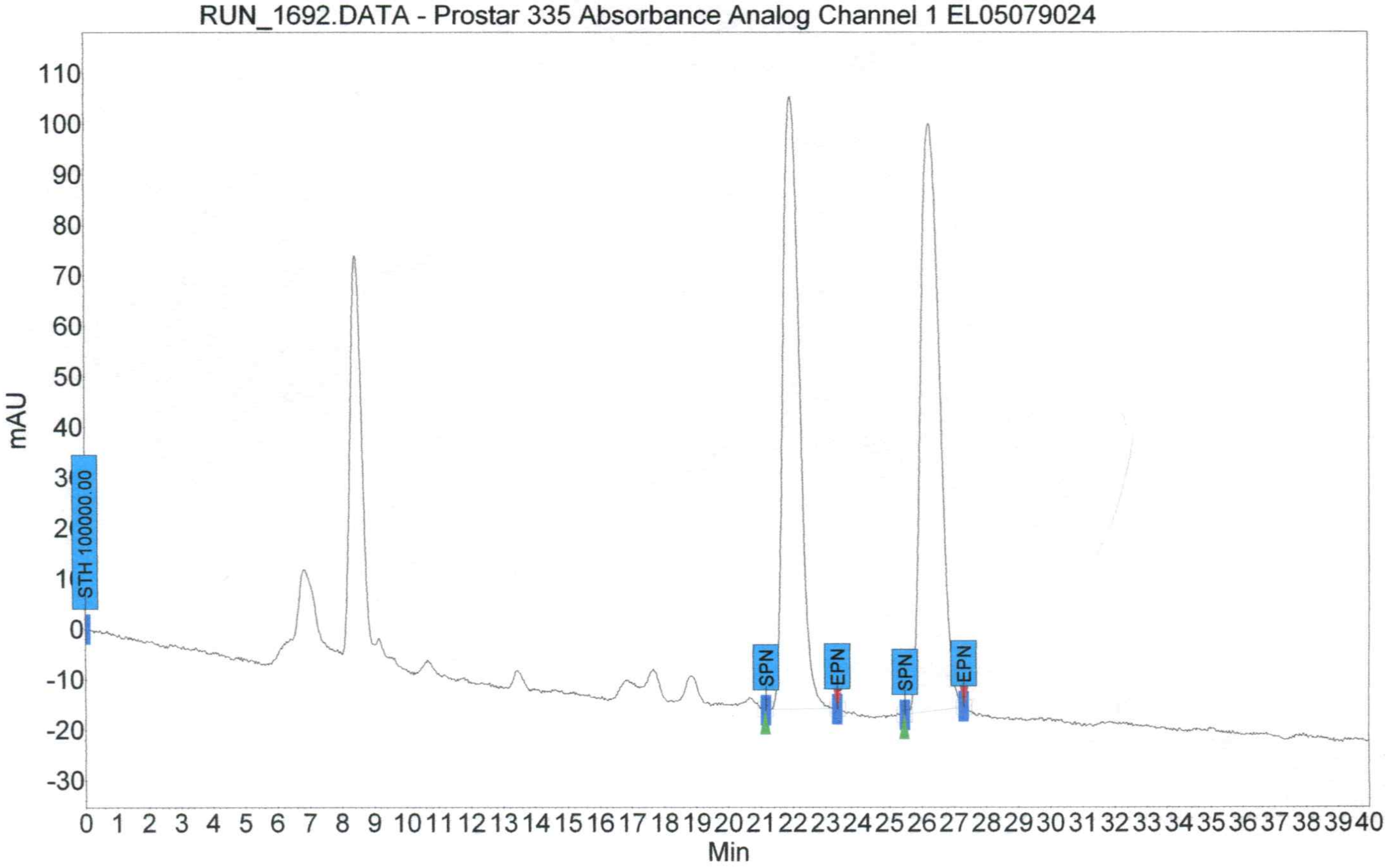

Index	Name	Time [Min]	Quantity [% Area]	Height [mAU]	Area [mAU.Min]	Area % [%]
1	UNKNOWN	21.92	47.55	121.3	71.7	47.546
2	UNKNOWN	26.23	52.45	116.4	79.1	52.454
Total			100.00	237.7	150.8	100.000

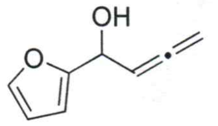

(±)-**2i**

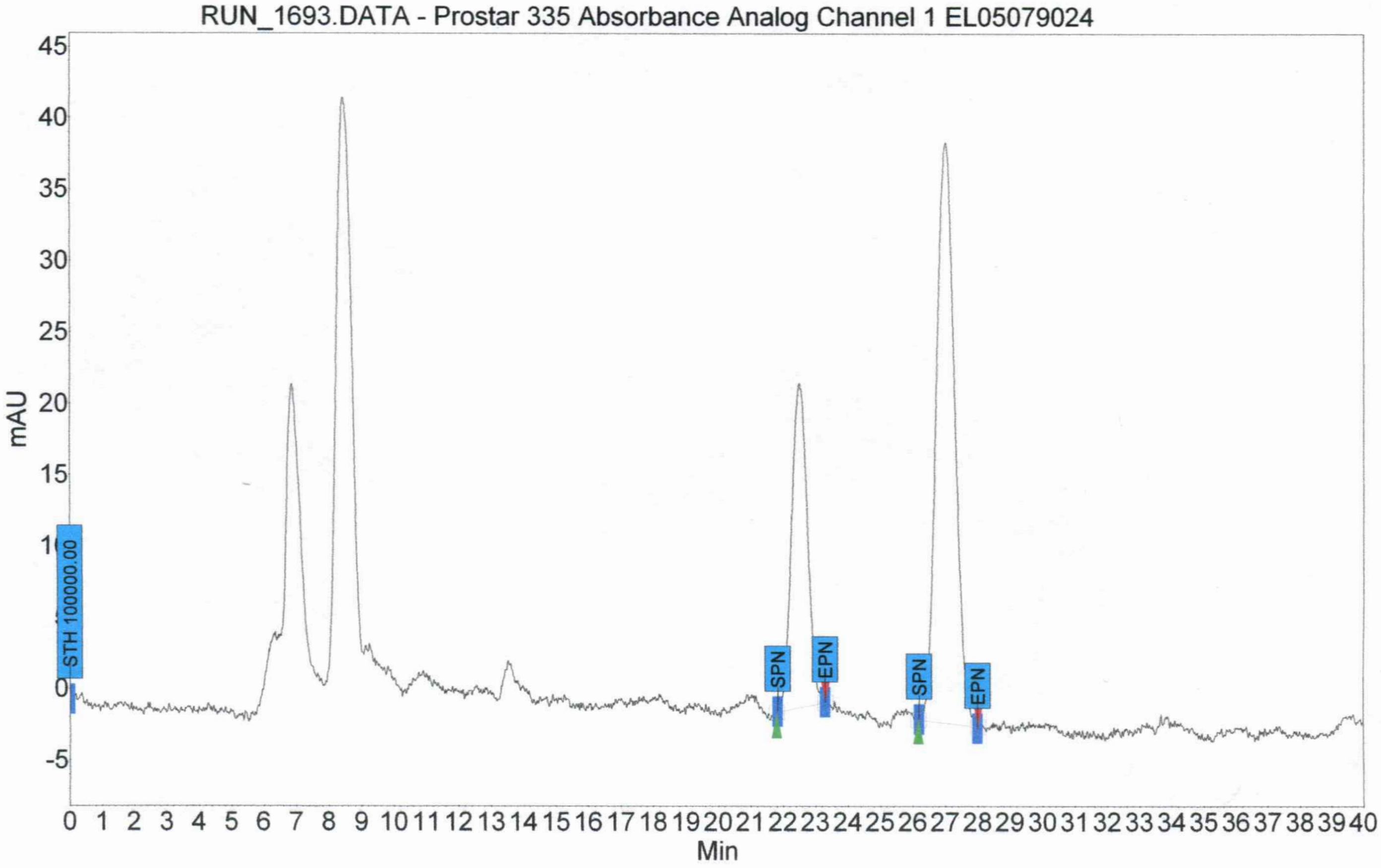

Index	Name	Time [Min]	Quantity [% Area]	Height [mAU]	Area [mAU.Min]	Area % [%]
1	UNKNOWN	22.49	32.47	22.8	12.9	32.468
2	UNKNOWN	27.00	67.53	40.7	26.8	67.532
Total			100.00	63.4	39.6	100.000

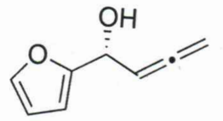

2*i*

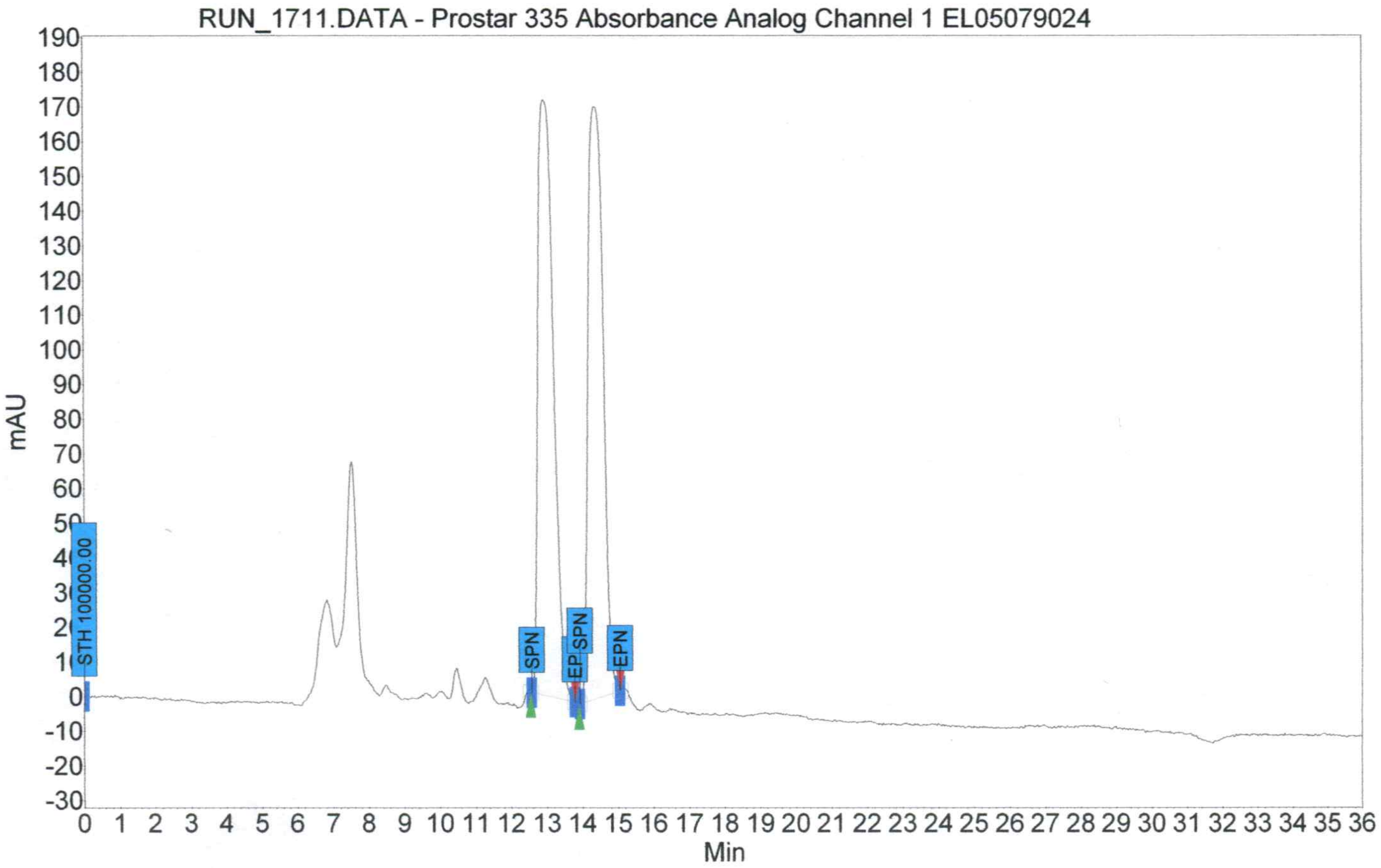

Index	Name	Time [Min]	Quantity [% Area]	Height [mAU]	Area [mAU.Min]	Area % [%]
1	UNKNOWN	12.93	50.86	171.7	86.4	50.861
2	UNKNOWN	14.33	49.14	170.9	83.5	49.139
Total			100.00	342.5	169.9	100.000

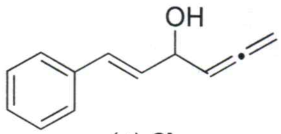

(±)-**2j**

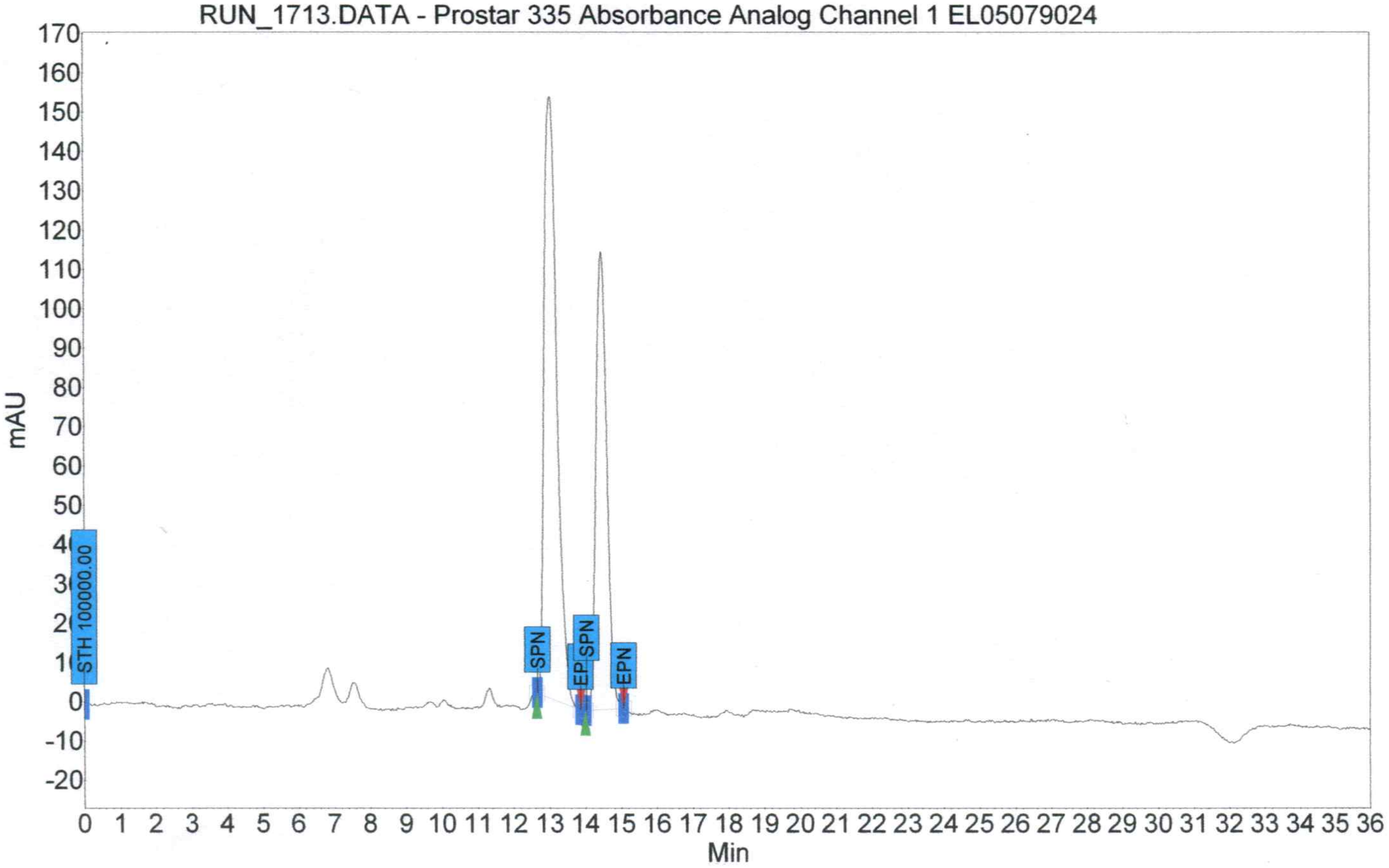

Index	Name	Time [Min]	Quantity [% Area]	Height [mAU]	Area [mAU.Min]	Area % [%]
1	UNKNOWN	13.03	60.65	153.1	62.9	60.653
2	UNKNOWN	14.45	39.35	116.6	40.8	39.347
Total			100.00	269.7	103.7	100.000

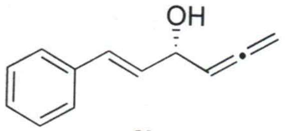

2j

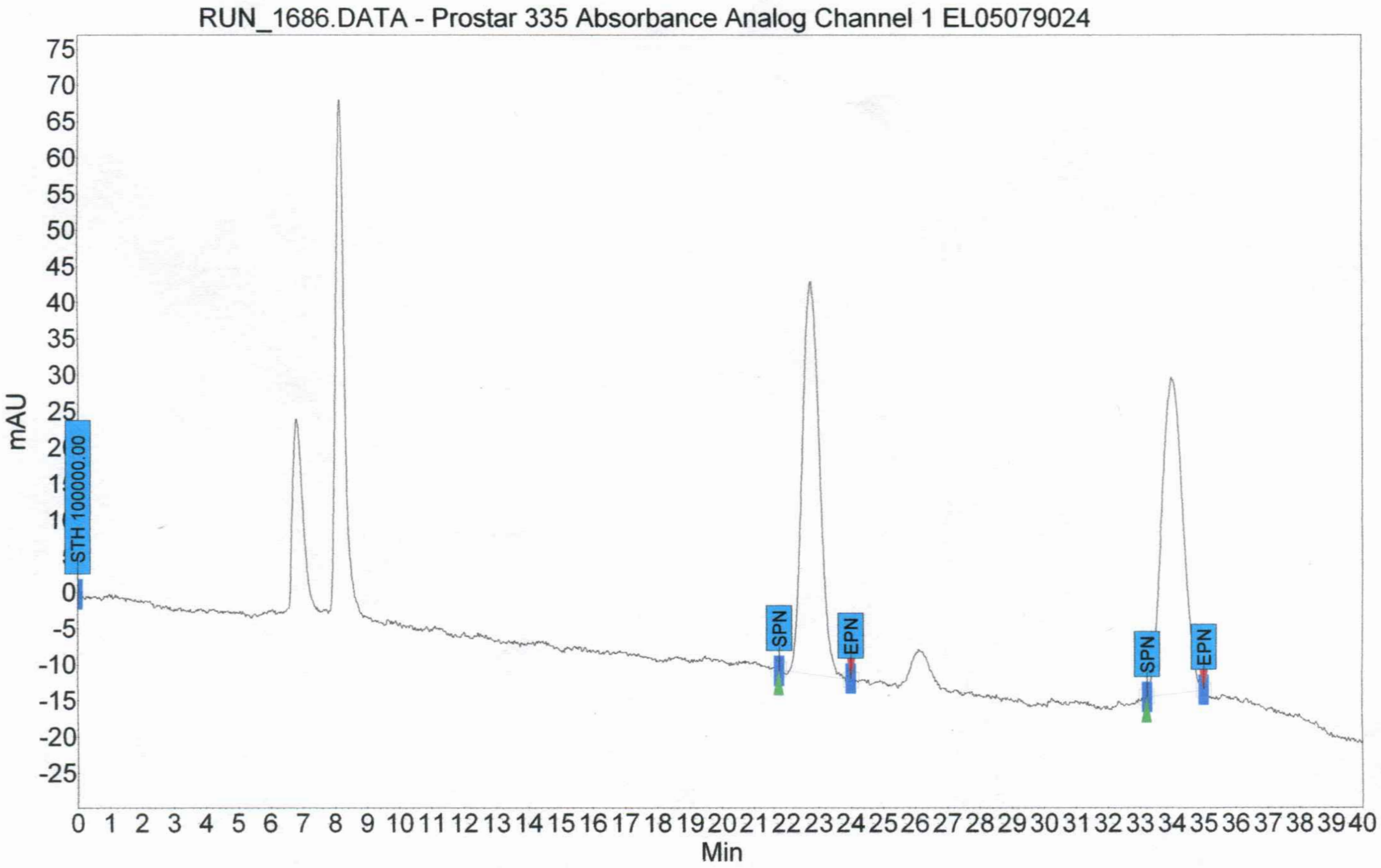

Index	Name	Time [Min]	Quantity [% Area]	Height [mAU]	Area [mAU.Min]	Area % [%]
1	UNKNOWN	22.76	50.04	54.3	33.4	50.041
2	UNKNOWN	33.99	49.96	43.7	33.3	49.959
Total			100.00	98.0	66.7	100.000

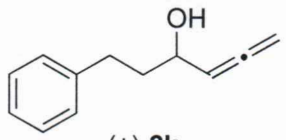

(±)-**2k**

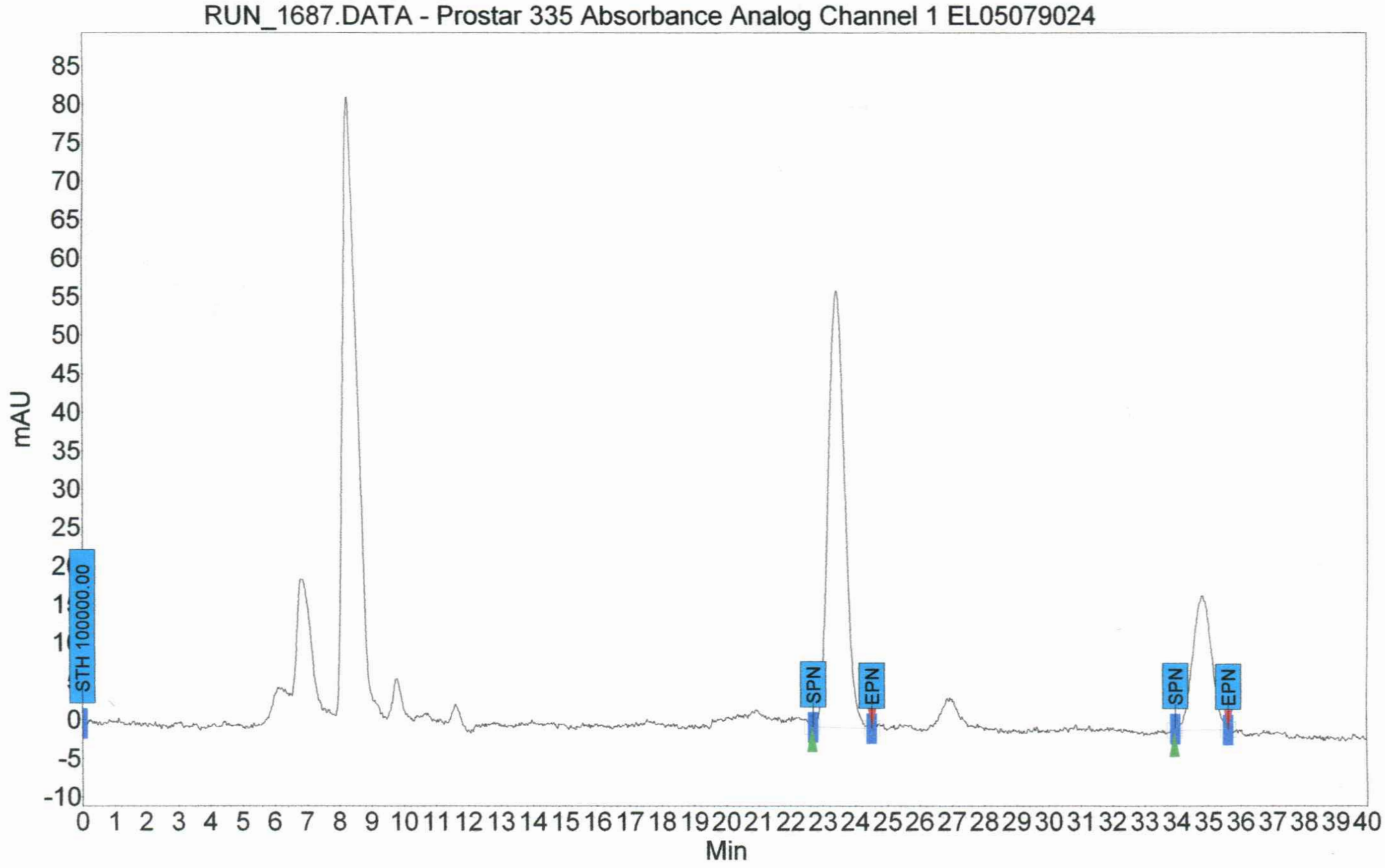

Index	Name	Time [Min]	Quantity [% Area]	Height [mAU]	Area [mAU.Min]	Area % [%]
1	UNKNOWN	23.40	73.88	56.7	33.7	73.875
2	UNKNOWN	34.79	26.12	17.3	11.9	26.125
Total			100.00	74.1	45.7	100.000

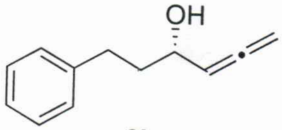

2k
